# Supplementary material for: Evolutionary analysis of chloroplast tRNA of Gymnosperm revealed the novel structural variation and evolutionary aspect
Source: PeerJ. 2020 Nov 25;8:e10312. doi: 10.7717/peerj.10312 (PMC7698693; doi:10.7717/peerj.10312)
Supplement: Supplemental Information 1 [file peerj-08-10312-s001.docx]

Data S1. tRNA sequences of gymnosperms chloroplast genome conducted in the study.

>trnH_GUG_118920_Welwitschia_mirabilis

GCGGACGTAGCCAAGTGGCtcAAGGCAGTGGATTGTGGATCCACCACgCGCGGGTTCAATCCCCGTCGTTCGCC

>trnC_GCA_34644_Welwitschia_mirabilis

GGCGACATGGCCAAGCGGTAAGGCAGGGGACTGCAAATCCTCGAtCCCCAGTTCAAATCTGGGTGTCGCCT

>trnL_UAG_90133_Welwitschia_mirabilis

GCCGCCATGGTGAAATAGGTaGACACGCTGCTCTTAGGAAGCAGTGCCAgAGcgTCTCGGTTCGAATCCGAGTGGCGGCA

>trnK_UUU_1145_Welwitschia_mirabilis

GGGTTGCTAACTCAATGGTAGAGTACTCGGCTTTTAACCGAAGAGtTCCGGGTTCGAATCCCGGGCAACCCA

>trnF_GAA_39040_Welwitschia_mirabilis

ACCAGGATAGCTCAGTTGGTAGAGCAGAGGACTGAAAATCCTCGTGtCACCAGTTCAATTCTGGTTCCTGGTA

>trnW_CCA_50083_Welwitschia_mirabilis

GCGCTCTTAGTTCAGCTtGGTAGAACGCAGGTCTCCAAAACCTGATGtCGTAGGTTCGAATCCTACAGGGCGTG

>trnE_UUC_105669_Welwitschia_mirabilis

GGGCTATTAGCTCAGCGGTAGAGCGCGCCCCTTTCACGGGCGAGGtCTCTGGTTCAAGTCCAGGATAGCCCA

>trnL_UAA_38388_Welwitschia_mirabilis

GGGGATATGGCGGAAGTGGTaGACGCTACGGACTTAAAATCCGTTGGTTTACaAGACTgTGAGGGTTCAAGTCCCTCTATCCCCA

>trnP_GGG_90317_Welwitschia_mirabilis

CGGGGTATAACGCAGATtGGCAGCGTGACATCATGGGGTGGTGTAAGtCACGGGTTCGATTCCCGTTGCTCCGA

>trnL_CAA_76801_Welwitschia_mirabilis

GCCTTGATGGTGAAATGGTaGACACGCGAGACTCAAAATCTCGTGCTAAAGAGCGTGGAGGTTCGAGTCCTCTTCAAGGCA

>trnR_UCU_34644_Welwitschia_mirabilis

GCGTCCATCGTCTAATGGAtAGGACAGAGGTCTTCTAAACCTTAAGTATAGGTTCAAGTCCTATTGGACGCA

>trnD_GUC_37752_Welwitschia_mirabilis

GGGATTGTAGTTCAATTGGTtAGAGTACCGCCCTGTCAAGACGGAAGtTGCGGGTTCGAGCCCCGTCAGTCCCG

>trnV_GAC_79656_Welwitschia_mirabilis

AGGGATATAACTCAGCGGGAGAGTGTCACCTTGACGTGGTGAAGGtCATCAGTTCGAGTCTGATTATCCCTA

>trnY_GUA_37523_Welwitschia_mirabilis

GGGTCGATGCCCGAGTGGTtaATGGGGACGGGCTGTAAACTCGTTGgtgTTAAtACCTACGCTGGTTCAAATCCAGCTCGGCCCA

>trnE_UUC_37389_Welwitschia_mirabilis

GCCCCTATCGTCTAGTGGCccAGGACATCTCTCTTTCAAGGAGGCAGCGGGGATTCGACTTCCCCTAGGGGTA

>trnR_CCG_44496_Welwitschia_mirabilis

GAGTTAGTAGCTCGGCGGAaaAGAGTTCATGGTTCCGGACCATAAGGtCAAGGGTTCAAATCCCTTCTAGCTCG

>trnT_GGU_5065_Welwitschia_mirabilis

GCCCTTTTAACTCAGTGGTAGAGTAACGCCATGGTAAGGCGTAAGtCATCGGTTCAAATCCGATAAAGGGCT

>trnS_UGA_8394_Welwitschia_mirabilis

GGAGAGATGGCCGAGTGGTttATGGCTCCGGTCTTGAAAACCGGCATAGTATCAAGCTATCGAGGGTTCGAATCCCTCTCTCTCCT

>trnR_CCG_48561_Welwitschia_mirabilis

GAGTTAGTAGCTCAGCGGAaaAGAGTTCATGGTTCCGGACCATGAGGtCAAGGGTTCAAATCCCTTCTAGCTCG

>trnT_UGU_38002_Welwitschia_mirabilis

GCCTGCTTAGCTCAGAGGTtAGAGCATCGCATTTGTAATGCGATGGtCATCGGTTCGATCCCGATAGCCGGCT

>trnI_CAU_68903_Welwitschia_mirabilis

GCATCCATGGCTGAATGGTtAAAGCGCCCAACTCATAATTGGcGAACtCGCAGGTTCAATTCCTGCTGGATGCA

>trnN_GUU_100730_Welwitschia_mirabilis

TCCCCAATAGCTCAGTGGTAGAGCGTTCGGCTGTTAACCGATTCGtCGTAGGTTCAAATCCTACTTGGGGAG

>trnA_UGC_82684_Welwitschia_mirabilis

GGGGATATAGCTCAGTTGGTAGAGCTCCGCTCTTGCATGGCGGATGtCAGCGGTTCGAGTCCGCTTATCTCCA

>trnV_UAC_39496_Welwitschia_mirabilis

AGGGCTATAGCTCAGTTGGTAGAGCACCTCGTTTACACCGAGAAGGtCTACGGTTCAAGCCCGTATAGCCCTA

>trnP_UGG_50230_Welwitschia_mirabilis

AGGGATGTAGCGCAGCTtgGGTAGCGCGTTTGTTTTGGGTACAAAATGtCGCAGGTTCAAATCCTGTCATCCCTA

>trnG_GCC_9009_Welwitschia_mirabilis

GCGGATATGGTTGAATGGTAAAATTTCTCTTTGCCAGGGAGAAGaCGCGGGTTCGATCCCCGCTATCCGCA

>trnM_CAU_9156_Welwitschia_mirabilis

TGCGGAGTAGAGTAGTCtGGTAGCTCGCAAGGCTCATACCCTTGAGGtCACGGGTTCAAATCCCGTCTTCGCGT

>trnR_ACG_87261_Welwitschia_mirabilis

GGGCCTGTAGCTCAGAGGAtaAGAGCACGTGGCTACGAACCACGGTGcCGGGGGTTCGAATCCCTCCTCGCCCA

>trnQ_UUG_37177_Welwitschia_mirabilis

TGGGGCGTCGCCAAGTGGTAAGGCAACAGGTTTTGGTCCTGTTATtCAAAGGTTCGAATCCTTTCGTCCCAG

>trnS_GCU_36132_Welwitschia_mirabilis

GGAGAGATGGCTGAGAGGAccAAAGCGACGGATTGCTAATCCGTTGTACGGGTAATCCCGTACCGAGGGTTCGAATCCCTCTCTCTCCG

>trnS_GGA_34874_Welwitschia_mirabilis

GCGGGTATAGTTTAGTGGTAaaagtgtgatttcgactatAACCCCTAGCCTTCCAAGCTAACGaTGCGGGTTCGATTCCCGCTACCCGCT

>trnM_CAU_40317_Welwitschia_mirabilis

ACCTACTTAACTCAGCGGTtAGAGTATCGCTTTCATACGGCGAGAGtCATTGGTTCAAATCCAATAGTAGGTA

>trnL_UAG_103246_Cunninghamia_lanceolata

GCCGCCATGGTGAAATTGGTaGACACGCTGCTCTTAGGAAGCAGTGCTAgAGcaTCTCGGTTCGAATCCGAGTGGCGGCA

>trnM_CAU_127080_Cunninghamia_lanceolata

ACCCACTTAACTCAGTGGTtAGAGTATCGCTTTCATACGGCGAGAGtCATTGGTTCAAATCCAATAGTAGGTA

>trnF_GAA_118115_Cunninghamia_lanceolata

GCCGGGATAGCTCAGTTGGTAGAGCAGAGGACTGAAAATCCTTGTGtCACCAGTTCAAATCTGGTTCCTGGCA

>trnT_GGU_42938_Cunninghamia_lanceolata

GCGGGTATAGTTTAGTGGTaAAAGTGtgATTCgtCCTTCCAAGCTAACGaTGCGGGTTCGATTCCCGCTACCCGCT

>trnE_UUC_51599_Cunninghamia_lanceolata

GGGCTATTAGCTCAGTGGTAGAGCGCGCCCCTTTCACGGGCGAGGtCTCTGGTTCAAGCCCAGGATAGCCCA

>trnR_UCU_42688_Cunninghamia_lanceolata

GCGTCCATCGTCTAATGGAtAGGACAGAGGTCTTCTAAACCTTAGGTATAGGTTCAAATCCTATTGGACGCA

>trnS_GGA_9091_Cunninghamia_lanceolata

GGAAAGATGGCTGAGCGGTtcAAGGCGTAGCATTGGAACTGCTATGTAGGCTTCTGTTTACCGAGGGTTCGAATCCCTCTCTTTCCG

>trnV_GAC_55579_Cunninghamia_lanceolata

AGGGATATAACTCAGTGGTAGAGTGTCACCTTGACGTGGTGAAAGtCATCAGTTCAAACCTGATTATCCCTA

>trnM_CAU_17473_Cunninghamia_lanceolata

CGCGGAGTAGAGCAGTTtGGTAGCTCGCAAGGCTCATAACCTTGAAGtCACGGGTTCAAATCCCGTCTCCGCAA

>trnE_UUC_22791_Cunninghamia_lanceolata

GCCCCTATCGTCTAGTGGCccAGGACATCTCTCTTTCAAGGAGGCAaCGGGGATTCGATTTCCCCTAGGGGTA

>trnS_GCU_44535_Cunninghamia_lanceolata

GGAGAGATGGCTGAGCGGActAAAGCGGTGGATTGCTAATCCGTTGTACAGACTATCTGTACCGAGGGTTCGAATCCCTCTCTCTCCG

>trnH_GUG_134766_Cunninghamia_lanceolata

GCGGACGTAGCCAAGTGGTtcaAAGGCAGTGGATTGTGAATCCACCACgCGCGGGTTCGATCCCCGTCGTTCGCC

>trnK_CUU_46383_Cunninghamia_lanceolata

TCCTCAATAGCTCAGTGGTAGAGCAATCGGCTCTTGACCGATTATTAGtCGTAGGTTCGAATCCTATTTGAGGAG

>trnP_GGG_103021_Cunninghamia_lanceolata

CGGAGTATGGCGCAGTTtGGTAGCGTGCCATCTTGGGGTGGTGGAGGtCGTGGGTTCAAATCCTGCTGCTCCGA

>trnN_GUU_46290_Cunninghamia_lanceolata

TCCTCAATAGCTCAGTGGTAGAGCGGTCGGCTGTTAACCGATTAGtCGTAGGTTCGAATCCTATTTGAGGAG

>trnA_UGC_50692_Cunninghamia_lanceolata

GGGGATATAGCTCAGTTGGTAGAGCTCCGCTCTTGCATGGCGGATGtCAGCGGTTCGAGTCCGCTTATCTCCA

>trnP_UGG_91064_Cunninghamia_lanceolata

AGGGATGTAGCGCAGCTtGGTAGCGCGTTTGTTTTGGGTACAAAATGtCGCAGGTTCAAATCCTGTCATCCCTA

>trnV_UAC_127319_Cunninghamia_lanceolata

AGGGCTATAGCTCAGCTaGGTAGAGCACCTCGTTTACACCGAGAAGGtCTACGGTTCGAGCCCGTATAGCCCTA

>trnT_UGU_7850_Cunninghamia_lanceolata

GCCTGCTTAGCTCAGAGGTtAGAGCGTCGCACTTGTAATGCGATGGtCATCGGTTCGATCCCGATAGCTGGCT

>trnY_GUA_22925_Cunninghamia_lanceolata

GGGTCGATGCCCGAGTGGCtaATGGGGACGGACTGTAAATTCGTTGGCAATATGCCTaCGCTGGTTCAAATCCAGCTCGGCCCA

>trnI_CAU_60811_Cunninghamia_lanceolata

GCATCCATGGCTGAATGGTaAAAGCACCCAACTCATAATTGGGAAGtCGCGGGTTCAATTCCTGCTGGATGCA

>trnG_UCC_22487_Welwitschia_mirabilis

GCCCTTTTAACTCAGCGGTAGAGTAACGCCATGGTAAGGCGTAAGtCATCGGTTCAAGCCCGATAAAGGGCT

>trnL_CAA_69095_Cunninghamia_lanceolata

GCCTTGATGGTGAAATGGTaGACACGCGAGACTCAAAATCTCGTGCTAAACAGCGTGGAGGTTCGAGTCCTCTTCAAGGCA

>trnW_CCA_90841_Cunninghamia_lanceolata

GCGCTCTTAGTTCAGTTtGGTAGAACGCAGGTCTCCAAAACCTGATGtCGTAGGTTCAAATCCTACAGAGCGTG

>trnQ_UUG_46041_Cunninghamia_lanceolata

TGGGGCGTGGCCAAGCGGTAAGGCAGCAGGTTTTGGTCCTGTTAtTtCGAAGGTTCGAATCCTTTCGTCCCAG

>trnD_GUC_23222_Cunninghamia_lanceolata

GGGATTGTAGTTCAATTGGTtAGAGTACCGCCCTGTCAAGACGGAAGtTGCGGGTTCGAGCCCCGTCAGTCCCG

>trnQ_UUG_7625_Cunninghamia_lanceolata

TGGGGCGTGGCCAAGCGGCggtAAGGCAGCAGGTTTTGATCCTGTTAtTtCGAAGGTTCGAATCCTTTCGTCCCAG

>trnG_GCC_17651_Cunninghamia_lanceolata

GCGGGTATGGTTGAATGGTAGAATTTCTCCTTGCCAAGGAGAAGaTGCGGGTTCGATTCCCGCTACCCGCC

>trnR_ACG_46913_Cunninghamia_lanceolata

GGGCCTGTAGCTCAGAGGAttAGAGCACGTGGCTACGAACCACGGTGtCGGGGGTTCAAATCCCTCCTCGCCCA

>trnC_GCA_26571_Cunninghamia_lanceolata

GGCGGCATAGCCAAGTGGTAAGGCAGGGGACTGCAAATCCTCCAtCCCCAGTTCAAATCCGGGTGTCGCCT

>trnL_UAA_117276_Cunninghamia_lanceolata

GGGGATATGGCGGAATCGGTaGACGCTACGGACTTAAAATCCGTTGGTTTTAtAGATCgTGAGGGTTCGAGTCCCTCTATCCCCA

>trnS_UGA_18394_Cunninghamia_lanceolata

GGAGAGATGGCCGAGTGGTtgATGGCTCCGGTCTTGAAAACCGGTATAGTTCAAAAACTATCGAGGGTTCGAATCCCTCTCTCTCCT

>trnK_UUU_1537_Cunninghamia_lanceolata

GGGTTGCTAACTCAATGGTAGAGTACTCGGCTTTTAACCGACGAGcTCCGGGTTCAAGTCCCGGGCAACCCA

>trnH_GUG_71194_Taxus_mairei

GCGGACGTAGCCAAGTGGTccaAAGGCAGTGGATTGTGAATCCACCACgCGCGGGTTCAATCCCCGTCGTTCGCC

>trnL_UAA_115126_Taxus_mairei

GGGGATATGGCGGAATTGGTaGACGCTACGGACTTAAAATCCGTTGGTTTTAtAAACCGTGAGGGTTCAAGTCCCTCTATCCCCA

>trnI_UAU_37361_Taxus_mairei

AGGGATATAACTCAGTAGTAGAGTGTCACCTTTATGTGGTGAAAGtCATCAGTTCAAACCTGATTATCCCTA

>trnG_GCC_105489_Taxus_mairei

GCGGGTATTGTTTAATGGAtAAAATTTATTCTTGCCAAGGATAAGaTGCGGGTTCGATTCCCGCTACCCGCC

>trnI_AAU_119109_Taxus_mairei

AGGGCTATAGCTCAGCTtGGTAGAGCACCTCAtaATAAGAAgtttgatctgttctaccGAGAAGGtCTACGGTTCGAGCCCGTATAGCCCTA

>trnF_GAA_115973_Taxus_mairei

GTCGGGATAGCTCAGTTGGTAGAGCAGAGGACTGAAAATCCTCGTGtCACCAGTTCAAATCTGGTTCCTGGCA

>trnD_GUC_99499_Taxus_mairei

GGGATTGTAGTTCAATTGGTtAGAGTACCGCCCTGTCAAGACGGAAGtTGCGGGTTCGAGCCCCGTCAGTCCCG

>trnT_UGU_114516_Taxus_mairei

GCCTGCTTAGCTCAGAGGTtAGAGCATCGCACTTGTAATGCGACGGtCATCGGTTCGATCCCGATAGAAGGCT

>trnW_CCA_14035_Taxus_mairei

GCGCTCTTAGTTCAGTGcGGTAGAACGCAGGTCTCCAAAACCTGATGcCGTAGGTTCAAATCCTACAGAGCGCG

>trnM_CAU_105676_Taxus_mairei

CGCGGAGTAGAGCAGTAtGGTAGCTCGCAAGGCTCATAACCTTGAAGtCACGGGTTCAAATCCCGTCTCCGCTA

>trnI_CAU_71583_Taxus_mairei

GCATCCATGGCTGAATGGTaAAAGCACCCAACTCATAATTGGGAAGtCGCGGGTTCAATTCCTGCTGGATGCA

>trnR_ACG_45150_Taxus_mairei

GGGCCTGTAGCTCAGAGGAttAGAGCACGTGGTTACGAACCACGGTGtCGGGGGTTCGAATCCCTCCTCGCCCA

>trnR_UCU_81144_Taxus_mairei

GCGTCCATCGTCTAATGGAtAGGACAGAGGTCTTCTAAACCTTAGGTATAGGTTCAAATCCTATTGGACGTA

>trnL_UAG_50656_Taxus_mairei

GCCGCCATGGTGAAATTGGTaGACACGCTGCTCTTAGGAAGCAGTGCTAgAGcaTCTCGGTTCGAATCCGAGTGGTGGCA

>trnN_GUU_45672_Taxus_mairei

TCCTCAGTAGCTCAGTGGTAGAGCGGTCGGCTGTTAACCGATTGGtCGTAGGTTCAAATCCTATTTGAGGAG

>trnK_UUU_73018_Taxus_mairei

GGGTTGTTAACTCAATGGTAGAGTACTCGGCTTTTAACCGACGAGtTCCGGGTTCAAGTCCCGGGCAACCCA

>trnL_CAA_31982_Taxus_mairei

GCCTTGATGGTGAAATGGTaGACACGCGAGACTCAAAATCTCGTGCTAAACAGCGTGGAGGTTCGAATCCTCTTCAAGGCA

>trnP_UGG_14185_Taxus_mairei

AGGGATGTAGCGCAGCTtGGTAGCGCGTTTGTTTTGGGTACAAAATGtCGCAGGTTCAAATCCTGTCATCCCTA

>trnY_GUA_99746_Taxus_mairei

GGGTCGATGCCCGAGTGGCtaATGGGGACGGACTGTAAATCCGTTGGCAATAtGCTTACGCTGGTTCAAATCCAGCTCGGCCCA

>trnM_CAU_119804_Taxus_mairei

CCCACTTAACTCAGTGGTtAGAGTATCGCTTTCATACGGCGAGAGtCATTGGTTCAAATCCAATAGTAGGA

>trnS_GCU_80259_Taxus_mairei

GGAGAGATGGCTGAGCGGActAAAGCGGTGGATTGCTAATCCGTTGTACAGACTATCTGTACCGAGGGTTCGAATCCCTCTTTCTCCG

>trnI_CAU_23714_Taxus_mairei

GCATCCATGGCTGAATGGTaAAAGCACCCAACTCATAATTGGGAAGtCGCGGGTTCAATTCCTGCTGGATGCA

>trnE_UUC_99898_Taxus_mairei

GCCCCTATCGTCTAGTGGCccAGGACATCTCTCTTTCAAGGAGGCAaCGGGGATTCGATTTCCCCTAGGGGTA

>trnT_GGU_100844_Taxus_mairei

GCACTTTTAACTCAGTGGTAGAGTAACGCCATGGTAAGGCGTAAGtCATCGGTTCAAGCCCGATAAAGGGCT

>trnS_UGA_104821_Taxus_mairei

GGAGAGATGGCCGAGTGGTtgATGGCTCCGGTCTTGAAAACCGGTATAGTTTTAaAAACTATCGAGGGTTCGAATCCCTCTCTCTCCT

>trnC_GCA_97135_Taxus_mairei

GGCGACATAGCCAAGTGGTAAGGCAGGGGACTGCAAATCCCCCAtCCCCAGTTCAAATCCGGGTGTCGCCT

>trnP_GGG_50424_Taxus_mairei

CGGAGCATAACGCAGTTtGGTAGCGTGCCATCTTGGGGTGATGGAGGtCGCGGGTTCAAATCCTGTTGCTCCGA

>trnQ_UUG_78840_Taxus_mairei

TGGGGCGTGGCCAAGCGGTAAGGCAACAGGTTTTGGTCCTGcTATTgCGAAGGTTCGAATCCTTTCGTCCCAG

>trnC_GCA_137519_Sciadopitys_verticillata

GGCGGCATAGCCAAGTGGTAAGGCATGAGACTGCAAATTTCCTAtCCCCAGTTCAAATCTGGGTGCCGCCT

>trnI_CAU_34364_Sciadopitys_verticillata

GCATCCATGGCTGAATGGTaAAAGCACCCGACTCATAATCGGGAAGtCGCGGGTTCAATTCCTGCTGGATGCA

>trnT_GGU_94443_Sciadopitys_verticillata

TCGAACGTAGCCAAGGAttaAGgcAGCAATAGTTTtggtcCTACTATTGCGAAGGTTCAAATCCTTTCGTTCAAA

>trnW_CCA_56651_Sciadopitys_verticillata

GCGCTCTTAGTTCAGTCcGGTAGAACCCAGGTCTCCAAAACCTGATGtCGTAGGTTCAAATCCTACAGAGCGTG

>trnN_GUU_8201_Sciadopitys_verticillata

TCCTCAATAGCTCAGTGGTAGAGCGGTCGGCTGTTAACCGATTGGtCGTAGGTTCAAATCCTACTTGGGGAG

>trnF_GAA_81419_Sciadopitys_verticillata

GCCGGGATAGCTCAGTTGGTAGAGCAGAGGACTGAAAATCCTCGTGtCACCAGTTCAAGTCTGGTTCCTGGTA

>trnE_UUC_13581_Sciadopitys_verticillata

GGGCTATTAGCTCAGTGGTAGAGCGCGCCCCTTTCACGGGCGAGGtCTCTGGTTCAAGTCCAGGATAGCCCA

>trnS_GGA_78768_Sciadopitys_verticillata

GGAAAGATGGCCGAGTGGTtcAAGGCGTAGCATTGGAACTGCTATATAAGCTTCTGTTTACCGAGGGTTCGAATCCCTCTCTTTCCG

>trnL_UAG_117202_Sciadopitys_verticillata

GCCGCCATGGTGAAATTGGTaGACACGCTGCTCTTAGGAAGCAGTGCGAGAGCGTCTCGGTTCGAATCCGAGTGGCGGCA

>trnR_CCG_90492_Sciadopitys_verticillata

GGGTTGGTAGCTCAGTGGAtcAGAGCGTATGGTTCCGGACCATGATGcCGGGGGTTCGAATCCCTTCTAACCCA

>trnA_UGC_12642_Sciadopitys_verticillata

GGGGATATAGCTCAGTGGGtAGAGCTCCGCTCTTGCATGGCGGATGtCAGCGGTTCGAGTCCGCTTATCTCCA

>trnQ_UUG_94370_Sciadopitys_verticillata

GGACGTAGCCAAGCGGTAAGGCAGCAGGTTTTGGTCCTGCTAtTgCGAAGGTTCGAATCCTTTCGTCCCAG

>trnS_UGA_69531_Sciadopitys_verticillata

GGAGAGATGGCCGAGTGGTtgATGGCTCCGGTCTTGAAAACCGGTATAGTTCAAAAACTATCGAGGGTTCGAATCCCTCTCTCTCCT

>trnM_CAU_85812_Sciadopitys_verticillata

ACCTACTTAACTCAGCGGTtAGAGTATCGCTTTCATACGGCGAGAGtCATTGGTTCAAATCCAATAGTAGGTA

>trnL_CAA_23855_Sciadopitys_verticillata

GCCTTGATGGTGAAATGGTaGACACGCGAGACTCAAAATTTCGTGCTAAACAGCGTGGAGGTTCGAGTCCTCTTCAAGGCA

>trnS_GCU_92815_Sciadopitys_verticillata

GGAGAGATGGCTGAGCGGAccAAAGCGGTGGATTGCTAATCCGTTGTACAGACTCTCTGTACCGAGGGTTCGAATCCCTCTCTCTCCG

>trnV_UAC_85008_Sciadopitys_verticillata

AGGGCTATAGCTCAGTAaGGTAGAGCGCCTCGTTTACACCGAGAAGGtCTACGGTTCGAGCCCGTATAGCCCTA

>trnP_UGG_56418_Sciadopitys_verticillata

AGGGATGTAGCGCAGCTtGGTAGCGTGTTTGTTTTGGGTACAAAATGtCGCAGGTTCAAATCCTGTCATCCCTA

>trnV_GAC_18221_Sciadopitys_verticillata

AGGGATATAACTCAGTGGTAGAGTGTCACTCTGACTTAGTGAAAGtCATCAGTTCGAGTCTGATTATCCCTA

>trnE_UUC_64546_Sciadopitys_verticillata

GCCCCTATCGTCTAGTGGCctAGGACATCTCTCTTTCAAGGAGGTAaCGGGGATTCGACTTCCCCTAGGGGTA

>trnG_GCC_70419_Sciadopitys_verticillata

GCGGGTATGGTTAAATGGTATAATTTCTCCTTGCCAGGGAGAAGaTGCGGGTTCGATCCCCGCTACCCGCC

>trnM_CAU_70619_Sciadopitys_verticillata

CGCGGAGTAGAGCAGTTtGGTAGCTCGCGAGGCTCATAACCTTGAGAtCACGGGTTCAAATCCCGTCTCCGCAA

>trnD_GUC_64130_Sciadopitys_verticillata

GGGATTGTAGTTCAATTGGTtAGAGTACCGCCCTGTCAAGACGGAAGtTGCGGGTTCGAGCCCCGTCAGTCCCG

>trnR_ACG_8524_Sciadopitys_verticillata

GGGCCTGTAGCTCAGAGGAttAGAGCACGTGGCTACGAACCACGGTGtCGGGGGTTCGAATCCCTCCTCGCCCA

>trnQ_UUG_94297_Sciadopitys_verticillata

TGGGGCGTGGCCAAGCGGTAAGGCAGCAGGTTTTGGTCCTGCTAtTgCGAAGGTTCGAATCCTTTCGTCCCAG

>trnK_UUU_1530_Sciadopitys_verticillata

GGGTTGCTAACTCAATGGTAGAGTACTCGGCTTTTAACCGATAAGcTCCGGGTTCGAGTCCCGGGCAACCCA

>trnT_GGU_65711_Sciadopitys_verticillata

GCCCTTTTAACTCAGCGGTAGAGTAACGCCATGGTAAGGCGTAAGtCATCGGTTCAACCCCGATAAAGGGCT

>trnH_GUG_120805_Sciadopitys_verticillata

GCGGACGTAGCCAAGTGGAtcAAGGCAGTGGATTGTGAATCCATCACgCGCGGGTTCAATCCCCGTCGTTCGCC

>trnY_GUA_64404_Sciadopitys_verticillata

GGGTCGATGCCCGAGTGGTtaATGGGGACGGACTGTAAATTCGTTGGCAATATGTCTaCGCTGGTTCAAATCCAGCTCGGCCCA

>trnL_UAA_80565_Sciadopitys_verticillata

GGGGATATGGCGGAATTGGTaGACGCTACGGACTTAAAATCCGTTGGTTTTCtAGACCgTGAGGGTTCAAGTCCCTCTATCCCCA

>trnR_UCU_90759_Sciadopitys_verticillata

ACGTCCATAGTCTAATGGAaAGGACAGAGGTCTTCTAAACCTTCGGTATAGGTTCGAATCCTATTGGACGTA

>trnP_GGG_72305_Retrophyllum_piresii

CGGGGCATGGCGCAGTTtGGTAGCGTGCCATCTTGGGGTGATGGAAGtCGTGGGTTCAAATCCCGCTGCTCCGA

>trnD_GUC_133138_Retrophyllum_piresii

GGGATTGTAGTTCAATTAGTtAGAGTACCGCCCTGTCAAGACGGAAGtTGCGGGTTCGAGCCCCGTCAGTCCCG

>trnM_CAU_21077_Retrophyllum_piresii

ACCCACTTAACTCAGTGGTtAGAGTATCGCTTTCATACGGCGAGAGtCATTGGTTCAAATCCAATAGTAGGTA

>trnR_CCG_25821_Retrophyllum_piresii

GGGTTGGTAGCTCAGTGGAtcAGAGCACATGGTTCCGGACCTTGGAGtCAAGGGTTCAAATCCCTTCTAGCCCG

>trnP_UGG_35886_Retrophyllum_piresii

AGGGATGTAGCGCAGCTtGGTAGCGCGTTTGTTTTGGGTACAAAATGtCGCAGGTTCAAATCCTGTCATCCCTA

>trnD_GUC_25574_Retrophyllum_piresii

GGGATTGTAGTTCAATTAGTtAGAGTACCGCCCTGTCAAGACGGAAGtTGCGGGTTCGAGCCCCGTCAGTCCCG

>trnT_UGU_15886_Retrophyllum_piresii

GCCTGCTTAGCTCAGAGGTtAGAGCATCGCACTTGTAATGCGATGGtCATCGGTTCGATCCCGATAGCTGGCT

>trnS_UGA_5103_Retrophyllum_piresii

GGAGAGATGGCCGAGTGGTtgATGGCTCCGGTCTTGAAAACCGGTATAGTTCAAAAACTATCGAGGGTTCGAATCCCTCTCTCTCCT

>trnK_UUU_82037_Retrophyllum_piresii

GGGTTGCTAACTCAATGGTAGAGTACTCGGCTTTTAACCGACGAGcTCCGGGTTCGAGTCCCGGGCAACCCA

>trnN_GUU_76577_Retrophyllum_piresii

TCCTCAGTAGCTCAGTGGTAGAGCGGTCGGCTGTTAACCGATTAGtCGTAGGTTCAAATCCTACTTGGGGAG

>trnL_CAA_97776_Retrophyllum_piresii

GCCTTGATGGTGAAATGGTaGACACGCGAGACTCAAAATCTCGTGCTATAAAGCGTGGAGGTTCGAGTCCTCTTCAAGGCA

>trnG_UCC_130291_Retrophyllum_piresii

GGCGACATGGCCAAGTGGTAAGGCAGGAGACTGCAAATCTCCCAtCCCCAGTTCGAATCTGGGTGTCGCCT

>trnY_GUA_152_Retrophyllum_piresii

GGGTCGATGCCCGAGTGGTtcATGGGGACGGACTGTAAATTCGTTGTGCAAtAtGCCGaCGCTGGTTCAAATCCAGCTCGACCCA

>trnV_GAC_103285_Retrophyllum_piresii

AGGGATATAACTCAGTGGTAGAGTATCACCTTGACGTGGTGATAGtCATCGGTTCGAATCCGATTATCCCTA

>trnS_GCU_77636_Retrophyllum_piresii

GGAGAGATGGCTGAGCGGAccAAAGCGGCGGATTGCTAATCCGTTGTACAGACTATCTGTACCGAGGGTTCGAATCCCTCTCTCTCCG

>trnF_GAA_17256_Retrophyllum_piresii

GCCGGGATAGCTCAGTTGGTAGAGCAGAGGACTGAAAATCCTCGTGcCACCAGTTCAAATCTGGTTCCTGGCA

>trnG_GCC_5971_Retrophyllum_piresii

GCGGGTATGGTTTAATGGTAAAATTTCTCTTTGCCAGGGAGAAGaTGCGGGTTCGATCCCCGCTACCCGCC

>trnL_UAA_16365_Retrophyllum_piresii

GGGGATATGGCGGAATTGGTaGACGCTACGGACTTAAAATCCGTCGgttttTTTAgACCTTGAGGGTTCAAGTCCCTCTATTCCCA

>trnL_UAG_72106_Retrophyllum_piresii

GCCGCCATGGTGAAATTGGTaGACACGCTGCTCTTAGGAAGCAGTGCTAgAGcaTCTCGGTTCGAATCCGAGTGGCGGCA

>trnW_CCA_35651_Retrophyllum_piresii

GCGCTCTTAGTTCAGTTcGGTAGAACGTGGGTCTCCAAAACCCGATGtCGTAGGTTCAAATCCTACAGAGCGTG

>trnQ_UUG_79005_Retrophyllum_piresii

TGGGGCGTGGCCAAGTGGTAAGGCAACAGGTTTTGGTCCTGTTAtTgCGAAGGTTCGAATCCTTCCGTCCCAG

>trnV_UAC_20310_Retrophyllum_piresii

AGGGCTATAGCTCAGCAaGGTAGAGCACCTCGTTTACACCGAGAAGGtCTACGGTTCGAGCCCGTATAGCCCTA

>trnI_CAU_90145_Retrophyllum_piresii

GCATCCATGGCTGAATGGTaAAAGCACCCAACTCATAATTGGGAAGtCGCGGGTTCAATTCCTGCTGGATGCA

>trnR_ACG_112715_Retrophyllum_piresii

GGGCCTGTAGCTCAGAGGAttAGAGCACGTGGTTACGAACCACGGTGtCGGGGGTTCAAATCCCTCCTGGCTCA

>trnH_GUG_86732_Retrophyllum_piresii

GTGGACGTCGCCAAGTGGTtAAGGCAGTGGATTGTGAATCCACCACgCGCGGGTTCAATCCCCGTCGTTCGCC

>trnC_GCA_108018_Retrophyllum_piresii

GGGGATATAGCTCAGTTGGTAGAGCTCCGCTCTTGCAAtggGCGGTGATTTACCCTATGGCGGATGtCAGCGGTTCGAGTCCGCTTATCTCCA

>trnS_GGA_113218_Retrophyllum_piresii

GCGGGTATAGTTTAGTGGTAaaagtgtgatttcgactatAACCCCTAGCCTTCCAAGCTAACGaTGCGGGTTCGATTCCCGCTACCCGCT

>trnE_UUC_106909_Retrophyllum_piresii

GGGCTATTAGCTCAGCGGTAGAGCGCGCCCCTTTCACGGGCGAGGaCTCTGGTTCAAGTCCAGGATAGCCCA

>trnT_GGU_1314_Retrophyllum_piresii

GCCCTTTTAACTCAGTGGTAGAGTAACGCCATGGTAAGGCGTAAGtCATCGGTTCAAGCCCGATAAAGGGCT

>trnE_UUC_306_Retrophyllum_piresii

GCCCCTATCGTCTAGTGGCccAGGACATCTCTCTTTCAAGGAGGCAaCGGGGATTCGACTTCCCCTAGGGGTA

>trnR_UCU_114287_Retrophyllum_piresii

GCGTCCATCGTCTAATGGAtAGGACAGAGGTCTTCTAAACCTTAGGTATAGGTTCGAATCCTATTGGACGTA

>trnM_CAU_6159_Retrophyllum_piresii

CGCGGGGTAGAGCAGTTtGGTAGCTCGCAAGGCTCATAACCTTGAAGtCACGGGTTCAAATCCCGTCTCCGCTA

>trnR_UCU_32616_Ephedra_equisetina

GCGTCCATCGTCTAATGGAtAGGACAGAGGTCTTCTAAACCTTAAaTATAGGTTCAAATCCTATTGGACGCA

>trnS_GCU_33456_Ephedra_equisetina

GGAGAGATGGCTGAGAGGAccAAAGCGGCGGATTGCTAATCCGTTGTACGGACAAtcCCGTATCGAGGGTTCGAATCCCTCTCTCTCCG

>trnW_CCA_40115_Ephedra_equisetina

GAGTTAGCAGCTCAGTGGAaaAGAGCCCACGGTTCCAGACCATGATGtCAAGGGTTCAACCCCCTTCTAGCTCA

>trnL_CAA_101987_Ephedra_equisetina

GCCTTGATGGTGAAATGGTaGACACGCGAGACTCAAAATCTCGTGCTaaGCGCGTGGAGGTTCGAGTCCTCTTCAAGGCA

>trnE_UUC_97046_Ephedra_equisetina

GGGCTATTAGCTCAGCGGTAGAGCGTGCCCCTTTCACGGGCGAGGTCGGGGtCTCTGGTTCAAGTCCAGGATAGCCCA

>trnR_ACG_77681_Ephedra_equisetina

GGGCCTGTAGCTCAGAGGAtaAGAGCACGTGGCTACGAACCACGGTGtCGGGGGTTCGAATCCCTCCTCGCCCA

>trnL_UAG_82595_Ephedra_equisetina

GCCGCCATGGTGAAATAGGTaGACACGCTGCTCTTAGGAAGCAGTGCTTGAGCtTCTCGGTTCGAGTCCGAGTGGCGGCA

>trnI_CAU_60791_Ephedra_equisetina

GCATCCATGGCTGAATGGTtAAAGCGCCCAACTCATAATTGGcGAACtCGCGGGTTCAATTCCTGCTGGATGCA

>trnM_CAU_8795_Ephedra_equisetina

CGCGGAGTAGAGTAGCCtGGTAGCTCGCAAGGCTCATAACCTTGAGGtCACGGGTTCAAATCCCGTCTCCGCCA

>trnH_GUG_61119_Ephedra_equisetina

GCGGACGTAGCCAAGTGGCtcAAGGCAGTGGATTGTGAATCCACCACgCGCGGGTTCAATCCCCGTCGTTCGCC

>trnC_GCA_17629_Ephedra_equisetina

GGCGGCATAGCCAAGTGGTAAGGCAGAGGACTGCAAATCCCTGATcCCCCAGTTCAAATCTGGGTGTCGCCT

>trnN_GUU_77973_Ephedra_equisetina

TCCCCAATAGCTCAGTGGTAGAGCGGTCGGCTGTTAACCGATTTTAGtCGTAGGTTCAAATCCTACTTGGGGAG

>trnS_GGA_16107_Ephedra_equisetina

GGAAAGATGGTCGAGTGGTtcAAGACGTAGCATTGGAAATGCTATAtAGACTTATGTCTAcCGAGGGTTCAAATCCCTCTCTTTCCG

>trnV_GAC_70330_Ephedra_equisetina

AGGGATATAACTCAGCGGGAGAGTGTCACCTTGACGTGGTGGAGGtCATCAGTTCGAGTCTGATTATCCCTA

>trnW_CCA_44586_Ephedra_equisetina

GCGCTCTTAGTTTAGCTtGGCAGAACGTAGGTCTCCAAAGCCTAATTcCATAGGTTCAAATCCTACAGAGCGTG

>trnY_GUA_34413_Ephedra_equisetina

GGGTCGATGCCCGAGTGGTtaATGGGGACGGGCTGTAAACTCGTTGgcATTAtACCTACGCTGGTTCAAATCCAGCTCGACCCA

>trnT_GGU_5370_Ephedra_equisetina

GCCCTTTTTACTCAGTGGTAGAGTAAAGCCATGGTAAGGCGTAAGtCATCGGTTCAAATCCGATAATGGACT

>trnQ_UUG_34120_Ephedra_equisetina

TGGGACGTCGCCAAGCGGTAAGGCAACAGGTTTTGGTCCTGTTATTACAAAGGTTCGAATCCTTTCGTCCCAG

>trnM_CAU_36161_Ephedra_equisetina

ACCTACTTAACTCAGTGGTtAGAGTATCGCTTTCATAAGGCGAGAGtCATTGGTTCAAATCCAATAGTAGGTA

>trnA_UGC_96165_Ephedra_equisetina

GGGGATATAGCTCAGTTGGTAGAGCTCCGCTCTTGCATGGCGGATGtCAGCGGTTCGAGTCCGCTTATCTCCA

>trnE_UUC_34302_Ephedra_equisetina

GCCCCTATCGTCTAGTGGCccAGGACATCTCTCTTTCAAGGAGGCAGCGGGGATTCGACTTCCCCTAGGGGTA

>trnP_UGG_44732_Ephedra_equisetina

AGGGATGTAGCGCAGCTtgGGTAGCGCGTTTGTTTTGGGTACAAAATGtCACAGGTTCAAATCCTGTCATCCCTA

>trnL_UAA_35050_Ephedra_equisetina

GGGGATATGGCGGAATTGGTaCACGCTACGGACTTAAAATCCGTTGGTCTACgAGACCtTGAGGGTTCAAGTCCCTCTATCCCCA

>trnF_GAA_35551_Ephedra_equisetina

GCCGGGATAGCTCAGTTGGTAGAGCAGAGGACTGAAAATTCTCGTGtCACCAGTTCAATTCTGGTTCCTGGTA

>trnS_UGA_8158_Ephedra_equisetina

GGAGAGATGGCCGAGTGGTttATGGCTCCGGTCTTGAAAACCGGCATAGGTTacaaACTATCGGGGGTTCGAATCCCTCTCTCTCCT

>trnD_GUC_34603_Ephedra_equisetina

GGGATTGTAGTTCAATTGGTtAGAGTACCGCCCTGTCAAGACGGAAGtTGCGGGTTCGAGTCCCGTCAGTCCCG

>trnG_GCC_8708_Ephedra_equisetina

GCGGATATGGTTGAATGGTAAAATTTCTCTTTGCCAAGGAGAAGaCGCGGGTTCGATTCCCGCTATCCGCC

>trnK_UUU_1209_Ephedra_equisetina

GGGTTGCTAACTCAATGGTAGAGTACTCGGCTTTTAACCGACTAGtTCCGGGTTCGAATCCCGGGCAACCCA

>trnF_GAA_77512_Gnetum_gnemon

GCCGGGATAGCTCAGTCGGTAGAGCAGAGGACTGAAAATCCTCGTGtCACCAGTTCAATTCTGGTTCCTGGTA

>trnH_GUG_1622_Gnetum_gnemon

GCGGACGTAGCCAAGTGGCtcAAGGCAGTGGATTGTGAATCCACCACgCGCGGGTTCAATCCCCGTCGTTCGCC

>trnG_UCC_111027_Gnetum_gnemon

GCCCTTTTAACTCAGCGGTAGAGTAACGCCATGGTAAGGCGTAAGtCATCGGTTCAACCCCGATAAAGGGCT

>trnE_UUC_35348_Gnetum_gnemon

GGGCTATTAGCTCAGCGGTAGAGCGCGCCCCTTTCACGGGCGAGGtCTCTGGTTCAAGTCCAGGATAGCCCA

>trnM_CAU_48648_Gnetum_gnemon

ACCTACTTAACTCAGTGGTtAGAGTATCGCTTTCATACGGCGAGAGtCATTGGTTCAAGTCCAATAGTAGGTA

>trnV_GAC_11709_Gnetum_gnemon

AGGGATATAACTCAGCGGGAGAGTGTCACCTTGACGTGGTGAAGGtCATCAGTTCGAGTCTGATTATCCCTA

>trnT_UGU_78449_Gnetum_gnemon

GCCTGCTTAGCTCAGAGGTtAGAGCATCGCATTTGTAATGCGATGGtCATCGGTTCGATCCCGATAGCAGGCT

>trnP_UGG_66942_Gnetum_gnemon

AGGGATGTAGCGCAGCTtGGAtAGCGCGTTTGTTTTGGGTACAAAATGtCGCAGGTTCAAATCCTGTCATCCCTA

>trnM_CAU_76264_Gnetum_gnemon

CGCGGAGTAGAGTAGTCtGGTAGCTCGCAAGGCTCATAACCTTGAGGtCACGGGTTCAAATCCCGTCTCCGCTA

>trnC_GCA_97818_Gnetum_gnemon

GGCGGCATAGCCAAGCGGTAAGGCGGAGGACTGCAAATCCCCGAtCCCCAGTTCAAATCTGGGTGTCGCCT

>trnE_UUC_78878_Gnetum_gnemon

GCCCCTATCGTCTAGTGGCccAGGACATCTCTCTTTCAAGGAGGCGGCGGGGATTCGACTTCCCCTAGGGGTA

>trnW_CCA_67124_Gnetum_gnemon

GCGCTCTTAGTTCAGCTtGGTAGAACGCAGGTCTCCAAAACCTGATGcCGTAGGTTCAAATCCTACAGAGCGTG

>trnL_CAA_8867_Gnetum_gnemon

GCCTTGATGGTGAAATGGTaGACACGCGAGACTCAAAATCTCGTGCTAAGGAGCGTGGAGGTTCGAGTCCTCTTCAAGGCA

>trnQ_UUG_79080_Gnetum_gnemon

TGGGGCGTCGCCAAGTGGTAAGGCAACAGGTTTTGGTCCTGTTAtTtCGAAGGTTCGAATCCTTTCGTCCCAG

>trnR_ACG_19249_Gnetum_gnemon

GGGCCTGTAGCTCAGAGGAtgAGAGCACGTGGCTACGAACCACGGTGtCGGGGGTTCAAATCCCTCCTCGCCCA

>trnV_UAC_76485_Gnetum_gnemon

AGGGCTATAGCTCAGTCGGTAGAGCACCTCGTTTACACCGAGAAGGtCTACGGTTCAAGCCCGTATAGCCCTA

>trnT_GGU_80858_Gnetum_gnemon

GCGGGTATAGTTTAGTGGTaaaagtGTGACTCGTCCTTCCAAGctAACGATGCGGGTTCGATTCCCGCTACCCGCT

>trnR_UCU_81775_Gnetum_gnemon

GCGCCCATCGTCTAATGGAtAGGACAGAGGTCTTCTAAACCTTGGGTATAGGTTCAATTCCTATTGGGCGTA

>trnY_GUA_78740_Gnetum_gnemon

GGGTCGATGCCCGAGCGGTtaATGGGGGCGGGCTGTAAACTCGTTGGCGTTAATGTCTaCGCTGGTTCAAATCCAGCTCGACCCA

>trnD_GUC_78589_Gnetum_gnemon

GGGATTGTAGTTCAATTGGTtAGAGTACCGCCCTGTCAAGACGGAAGtTGCGGGTTCGAGCCCCGTCAGTCCCG

>trnS_UGA_107957_Gnetum_gnemon

GGAGAGATGGCCGAGTGGTttATGGCTCCGGTCTTGAAAACCGGCATGGTGAAAACTATCGAGGGTTCGAATCCCTCTCTCTCCT

>trnR_CCG_72128_Gnetum_gnemon

GAGTTAGTAGCTCAGCGGAgaAGAGCACATGGTTCCGGACCATGAGGtCAAGGGTTCAAATCCCTTCTAGCTCA

>trnK_UUU_112476_Gnetum_gnemon

GGGTTGCTAACTCAATGGTAGAGTACTCGGCTTTTAACCGAAGAGtTCCGGGTTCGAATCCCGGGCAACCCA

>trnP_GGG_27701_Gnetum_gnemon

TGGGGTATAACGCAGCTtGGTAGCGTGACATCATGGGGTGGTGTAAGtCGCGGGTTCAACTCCCGCTACCCCAA

>trnS_GCU_80232_Gnetum_gnemon

GGAGAGGTGGCTGAGAGGActAAAGCGGCGGTTTGCTAATCCGTTGTACGGATAAtcCCGTATCGAGGGTTCGAATCCCTCTCTCTCCG

>trnL_UAG_27863_Gnetum_gnemon

GCCGCCATGGTGAAATAGGTaGACACGCTGCTCTTAGGAAGCAGTGCCAgAGcaTCTCGGTTCGAATCCGAGTGGCGGCA

>trnG_GCC_107307_Gnetum_gnemon

GCGGATATGGTTGAATGGTAAAATTTCTCTTTGCCAGGGAGAAGaCGCGGGTTCGATCCCCGCTATCCGCA

>trnS_GGA_99522_Gnetum_gnemon

GGAAAGATGGCCGAGCGGTtcAAGGCGTAGCATCGGAACTGCTATGTAGACTTATGTCTACCGAGGGTTCGAATCCCTCTCTTTCCG

>trnN_GUU_19530_Gnetum_gnemon

TCCCCAATAGCTCAGCGGTAGAGCAATCGGCTGTTAACCGATTTTAGtCGTAGGTTCAAATCCTACTTGGGGAG

>trnL_UAA_77723_Gnetum_gnemon

GGGGATATGGCGGAATTGGTaGACGCTACGGACTTAAAATCCGTTGGTTTACgAAACTgTGAGGGTTCAAGTCCCTCTATCCCCA

>trnA_UGC_34443_Gnetum_gnemon

GGGGATATAGCTCAGTTGGTAGAGCTCCGCTCTTGCATGGCGGATGtCAGCGGTTCGAGTCCGCTTATCTCCA

>trnI_CAU_1264_Gnetum_gnemon

GCATCCATGGCTGAATGGTtAAAGCGCCCAACTCATAATTGGcGAACtCGCGGGTTCAATTCCTGCTGGATGCA

>trnT_GGU_129602_Wollemia_nobilis

GCCCTTTTAACTCAGTGGTAGAGTAACGCCATGGTAAGGCGTAAGtCATCGGTTCAAGCCCGATAAAGGGCT

>trnW_CCA_90887_Wollemia_nobilis

GCGCTCTTAGTTCAGTTcGGTAGAACGTAGGTCTCCAAAACCTGATGtCGTAGGTTCAAATCCTACAGAGCGTG

>trnV_GAC_36714_Wollemia_nobilis

AGGGATATAACTCAGCGGTAGAGTGTCACCTTGACGTGGTGATGGtCATCGGTTCGAGTCCGATTATCCCTA

>trnG_GCC_124623_Wollemia_nobilis

GCGGGTATGGTTGAATGGTAAAATTTCTCTTTGCCAGGGAGAAGaTGCGGGTTCGATCCCCGCTACCCGCC

>trnR_CCG_103032_Wollemia_nobilis

GGGTTGGTAGCTCAGTGGAtcAGAGCGCATGGTTCCGGACCTTGAAGtCAAGGGTTCGAATCCCTTCTAGCCCG

>trnS_CGA_6512_Wollemia_nobilis

GCGGGTATAGTTTAGTGGTAaaagtgtgatttcgactatAACCCCTAGCCTTCCAAGCTAACGaTGCGGGTTCGATTCCCGCTACCCGCT

>trnP_UGG_91130_Wollemia_nobilis

AGGGATGTAGCGCAGCTtGGTAGCGCGTTTGTTTTGGGTACAAAATGcCGCAGGTTCAAATCCTGTCATCCCTA

>trnI_CAU_21954_Wollemia_nobilis

GCATCCATGGCTGAATGGTcAAAGCGCCCAACTCATAATTGGGAAGtCGCGGGTTCAATTCCTGCTGGATGCA

>trnP_GGG_52968_Wollemia_nobilis

CGGGGCATGGCGCAGTTtGGTAGCGTGCCATCTTGGGGTGATGGAGGtCGCAGGTTCAAATCCCGCTGCTCCGA

>trnR_ACG_46496_Wollemia_nobilis

GGGCCTGTAGCTCAGAGGAttAGAGCACGTGGTTACGAACCACGGTGtCGGGGGTTCAAATCCCTCCTGGCCCA

>trnF_GAA_113114_Wollemia_nobilis

GCCGGGATAGCTCAGTTGGTAGAGCAGAGGACTGAAAATCCTCGTGcCACCAGTTCAAATCTGGTTCCTGGCA

>trnC_GCA_134757_Wollemia_nobilis

GGCGACATAGCCAAGTGGTAAGGCAGGGGACTGCAAATCCCCCAtCCCCAGTTCAAATCTGGGTGTCGCCT

>trnK_UUU_13684_Wollemia_nobilis

GGGTTGCTAACTCAATGGTAGAGTACTCGGCTTTTAACCGACGAGcTCCGGGTTCGAGTCCCGGGCAACCCA

>trnS_GGA_115942_Wollemia_nobilis

GGAAAGATGGCCGAGTGGTtgAAGGCGTAGCATTGGAACTGCTATGTAGGCTTATGTTTACCGAGGGTTCGAATCCCTCTCTTTCCG

>trnL_UAG_53167_Wollemia_nobilis

GCCGCCATGGTGAAATTGGTaGACACGCTGCTCTTAGGAAGCAGTGCTAgAGcaTCTCGGTTCGAATCCGAGTGGCGGCA

>trnM_CAU_124427_Wollemia_nobilis

TGCGGGGTAGAGCAGCTtGGTAGCTCGCGAGGCTCATAACCTTGAGGtCACGGGTTCAAATCCCGTCTCCGCTA

>trnL_UAA_113548_Wollemia_nobilis

GGGGATATGGCGGAATTGGTaGACGCTACGGACTTAAAATCCGTCGGTTTTTtAGACCgTGAGGGTTCAAGTCCCTCTATTCCCA

>trnS_UGA_125484_Wollemia_nobilis

GGAGAGATGGCCGAGTGGTtgATGGCTCCGGTCTTGAAAACCGGTATAGTTAaACTATCGAGGGTTCGAATCCCTCTCTCTCCT

>trnA_UGC_41240_Wollemia_nobilis

GGGGATATAGCTCAGTTGGTAGAGCTCCGCTCTTGCATGGCGGATGtCAGCGGTTCGAGTCCGCTTATCTCCA

>trnN_GUU_47307_Wollemia_nobilis

TCCTCAGTAGCTCAGTGGTAGAGCGGTCGGCTGTTAACTGACTGGtCGTAGGTTCAAATCCTACTTGGGGAG

>trnG_UCC_90178_Wollemia_nobilis

GCAAGTATGGATGGAaggAAATATTAGCCTTCCAAGCTAACGaTGCGGGTTCGATTCCCGCTACCCGCT

>trnH_GUG_18545_Wollemia_nobilis

GCGGACGTAGCCAAGTGGAtcAAGGCAGTGGATTGTGAATCCACCACgCGCGGGTTCAATCCCCGTCGTTCGCC

>trnR_UCU_6259_Wollemia_nobilis

GCGTCCATCGTCTAATGGAtAGGACAGAGGTCTTCTAAACCTCCGGTATAGGTTCAAATCCTATTGGACGTA

>trnM_CAU_107496_Wollemia_nobilis

ACCCACTTAACTCAGTGGTtAGAGTGTCGCTTTCATACGGCGAGAGtCATTGGTTCAAATCCAATAGTAGGTA

>trnQ_UUG_9806_Wollemia_nobilis

TGGGGCGTGGCCAAGCGGTAAGGCAGCAGGTTTTGGTCCTGTTAtTtCGAGGGTTCGAATCCTTCCGTCCCAG

>trnD_GUC_71649_Wollemia_nobilis

GGGATTGTAGTTCAATTGGTtAGAGTACCGCCCTGTCAAGACGGAAGtTGCGGGTTCGAGCCCCGTCAGTCCCG

>trnE_UUC_40148_Wollemia_nobilis

GCCCCTATCGTCTAGTGGCccAGGACATCTCTCTTTCAAGGAGGCAaCGGGGATTCGACTTCCCCTAGGGGTA

>trnT_UGU_114545_Wollemia_nobilis

GCCTGCTTAGCTCAGAGGTtAGAGCATCGCACTTGTAATGCGATGGtCATCGGTTCGATCCCGATAGCTGGCT

>trnL_CAA_30036_Wollemia_nobilis

GCCTTGATGGTGAAATGGTaGACACGCGAGACTCAAAATCTCGTGCTATAAAGCGTGGAGGTTCGAGTCCTCTTCAAGGCA

>trnY_GUA_107737_Wollemia_nobilis

AGGGCTATAGCTCAGCAaGGTAGAGCACCTCGTTtataataagaactttgatctgtTTTACCGAGAAGGtCTACGGTTCGAGCCCGTATAGCCCTA

>trnS_GCU_8172_Wollemia_nobilis

GGAGAGATGGCTGAGTGGActAAAGCGGCGGATTGCTAATCCGTTGTACAGACTATCTGTACCGAGGGTTCGAATCCCTCTCTCTCCG

>trnE_UUC_130551_Wollemia_nobilis

GGGCTATTAGCTCAGCGGTAGAGCGCGCCCCTTTCACGGGCGAGGtCTCTGGTTCAAGTCCAGGATAGCCCA

>trnF_GAA_16836_Cedrus_deodara

GCCGGGATAGCTCAGTTGGTAGAGCAGAGGACTGAAAATCCTCGTGcCACCAGTTCAAATCTGGTTCCTGGCA

>trnH_GUG_69151_Cedrus_deodara

GCGGACGTAGCCAAGTGGAccAAGGCAGTGGATTGTGAATCCACCACgCGCGGGTTCAATTCCCGTCGTTCGCC

>trnD_GUC_98431_Cedrus_deodara

GGGATTGTAGTTCAATTGGTtAGAGTACCGCCCTGTCAAGACGGAAGtTGCGGGTTCGAGCCCCGTCAGTCCCG

>trnC_GCA_96061_Cedrus_deodara

GGCGACATAGCCAAGCGGTAAGGCAGAGGACTGCAAATCCTCCAtCCCCAGTTCGAATCCGGGTGTCGCCT

>trnN_GUU_41931_Cedrus_deodara

TCCTCAGTAGCTCAGTGGTAGAGCGGTCGGCTGTTAACTGATTGGtCGTAGGTTCGAATCCTACTTGGGGAG

>trnL_UAG_54755_Cedrus_deodara

GCCGCCATGGTGAAATTGGTaGACACGCTGCTCTTAGGAAGCAGTGCGAGAGCATCTCGGTTCAAATCCGAGTGGCGGCA

>trnA_UGC_36537_Cedrus_deodara

GGGGATATAGCTCAGTTGGTAGAGCTCCGCTCTTGCATGGCGGATGtCAGCGGTTCGAGTCCGCTTATCTCC

>trnR_UCU_51510_Cedrus_deodara

GCGTCCATCGTCTAATGGAtAGGACAGAGGTCTTCTAAACCTCCGGTATAGGTTCAAATCCTATTGGACGTA

>trnG_GCC_28238_Cedrus_deodara

GCGGGTATGGTTGAATGGTAAAATTTCTCTTTGCCAAGGAGAAGaTGCGGGTTCGATCCCCGCTACCCGCC

>trnM_CAU_69483_Cedrus_deodara

ACCCACTTAACTCAGTGGTtAGAGTATCGCTTTCATACGGCGAGAGtCATTGGTTCAAATCCAATAGTAGGTA

>trnV_GAC_57402_Cedrus_deodara

AGGGATATAACTCAGCGGTAGAGTATCACCTTGACGTGGTGGAAGtCATCAGTTCGAGCCTGATTATCCCTA

>trnE_UUC_35405_Cedrus_deodara

GGGCTATTAGCTCAGTGGTAGAGCGCGCCCCTTTCACGGGCGAGGtCTCTGGTTCAAGTCCAGGATAGCCCA

>trnM_CAU_28008_Cedrus_deodara

TGCGGAGTAGAGTAGTCtGGTAGCTCGCAAGGCTCATAACCTTGAGGtCACGGGTTCGAATCCTGTCTCCGCCA

>trnS_GGA_78705_Cedrus_deodara

GCGGGTATAGTTTAGTGGTAaaagtgtgatttcgactatAACCCCTAGCCTTCCAAGCTAACGaTGCGGGTTCGATTCCCGCTACCCGCT

>trnI_CAU_15398_Cedrus_deodara

GCATCCATGGCTGAATGGTtAAAGCACCCAACTCATAATTGGcGAACtCGCGGGTTCAATTCCTGCTGGATGCA

>trnW_CCA_115364_Cedrus_deodara

GCGCTCTTAGTTCAGTTcGGTAGAACGTAGGTCTCCAAAACCTGATGcCGTAGGTTCAAATCCTACAGAGCGTG

>trnE_UUC_98926_Cedrus_deodara

GCCCCTATCGTCTAGTGGAccAGGACATCTCTCTTTCAAGGAGGCAGCGGGGATTCGACTTCCCCTAGGGGTACCA

>trnT_GGU_100375_Cedrus_deodara

GCCCTTTTAACTCAGCGGTAGAGTAACGCCATGGTAAGGCGTAAGtCATCGGTTCAAGTCCGATAAAGGGCT

>trnL_CAA_61986_Cedrus_deodara

GCCTTGATGGTGAAATGGTaGACACGCGAGACTCAAAATCTCGTGCTTAAGAGCGTGGAGGTTCGAGTCCTCTTCAAGGCA

>trnP_UGG_115611_Cedrus_deodara

AGGGATGTAGCGCAGCTtGGTAGCGTGTTTGTTTTGGGTACAAAATGtCGCAGGTTCAAATCCTGTCATCCCTA

>trnR_CCG_106734_Cedrus_deodara

GGGTTGGTAGCTCAGTGGAtcAGAGCCCATGGTTCCGGACCATGAAGtCAAGGGTTCAAATCCCTTCTAGCCCA

>trnY_GUA_101419_Cedrus_deodara

AGGGCTATAGCTCAGCAaGGTAGAGCACCTCGTTtataataagaactttgatctgtTCTACCGAGAAGGtCTACGGTTCGAGCCCGTATAGCCCTA

>trnG_UCC_98774_Cedrus_deodara

GGGTCGATGCCCGAGTGGTtaATGGGGGCGGACTGTAAATCCGTTGGCAATATGCCTaCGCTGGTTCAAATCCAGCTCGACCCA

>trnT_UGU_18275_Cedrus_deodara

GCCTGCTTAGCTCAGAGGTtAGAGCATCGCACTTGTAATGCGATGGtCATCGGTTCGATCCCGATAGCTGGCT

>trnP_GGG_54990_Cedrus_deodara

CGGAGCATGGCGCAGCTtGGTAGCGTGCCATCTTGGGGTGATGGAGGtCGTGGGTTCAGATCCCGCTGCTCCGA

>trnK_UUU_71308_Cedrus_deodara

GGGTTGCTAACTCAATGGTAGAGTACTCGGCTTTTAACCGACGAGcTCCGGGTTCGAGTCCCGGGCAACCCG

>trnL_UAA_17278_Cedrus_deodara

GGGGATATGGCGGAATTGGTaGACGCTACGGACTTAAAATCCGTTGgctTTAtAgACCGTGAGGGTTCAAGTCCCTCTATCCCCA

>trnS_GCU_1021_Cedrus_deodara

GGAGAGATGGCTGAGCGGAccAAAGCGGCGGATTGCTAATCCGTAGTACGGATCATCCGTACCGAGGGTTCGAATCCCTCTCTCTCCG

>trnS_UGA_29035_Cedrus_deodara

GGAGAGATGGCCGAGTGGTtgATGGCTCCGGTCTTGAAAACCGGTATAGTAAaaaaACTATCGAGGGTTCGAATCCCTCTCTCTCCT

>trnR_ACG_41120_Cedrus_deodara

GGGCCTGTAGCTCAGAGGAttAGAGCACGTGGCTACGAACCACGGTGtCGGGGGTTCGAATCCCTCCTCGCCCA

>trnQ_UUG_76449_Cedrus_deodara

TGGGGCGTGGCCAAGCGGTAAGGCAGCAGGTTTTGATCCTGTTATtCGGAGGTTCGAATCCTTCCGTCCCAG

>trnN_GUU_138071_Cycas_debaoensis

TCCTCAGTAGCTCAGTGGTAGAGCGGTCGGCTGTTAACTGACTGGtCGTAGGTTCAAATCCTACTTGGGGAG

>trnE_UUC_143585_Cycas_debaoensis

GGGCTATTAGCTCAGTGGTAGAGCGCGCCCCTTTCACGGGCGAGGtCTCTGGTTCAAGTCCAGGATAGCCCA

>trnF_GAA_48422_Cycas_debaoensis

GCCGGGATAGCTCAGTTGGTAGAGCAGAGGACTGAAAATCCTCGTGcCACCAGTTCAAATCTGGTTCCTGGCA

>trnS_UGA_36209_Cycas_debaoensis

GGAGAGATGGCCGAGTGGTtcATGGCTCCGGTCTTGAAAACCGGTAtggttaCAAAaAACTAtCGAGGGTTCGAATCCCTCTCTCTCCT

>trnD_GUC_31566_Cycas_debaoensis

GGGATTGTAGTTCAATTGGTcAGAGTACCGTCCTGTCAAGACGGAAGtTGCGGGTTCGAGTCCCGTCAGTCCCG

>trnS_GGA_45685_Cycas_debaoensis

GGAAAGATGGCCGAGCGGTtcAAGGCGTAGCACTGGAACTGCTATGTAGACTTTTGTTTACCGAGGGTTCGAATCCCTCTCTTTCCG

>trnL_UAA_47471_Cycas_debaoensis

GGGGATATGGCGGAATTGGTaGACGCTACGGACTTAAAATCCGTCGgctTTAtAgACCGTGAGAGTTCAAATCTCTCTATCCCCA

>trnT_UGU_10684_Cycas_debaoensis

GCGGGTATAGTTTAGTGGTaAAAGTGtgATTCgtCCTTCCAAGCTAACGaTGCGGGTTCGATTCCCGCTACCCGCT

>trnE_UUC_32087_Cycas_debaoensis

GCCCCTATCGTCTAGTGGCccAGGACATCTCTCTTTCAAGGAGGCAGCGGGGATTCGACTTCCCCTAGGGGTA

>trnY_GUA_31939_Cycas_debaoensis

GGGTCGATGCCCGAGTGGTtaATGAGGACGGACTGTAAATTCGTTGGCAATATGCCTaCGCTGGTTCAAATCCAGCTCGACCCA

>trnL_UAG_118790_Cycas_debaoensis

GCCGCCATGGTGAAATTGGTaGACACGCTGCTCTTAGGAAGCAGTGCTAggGCaTCTCGGTTCGAATCCGAGTGGCGGCA

>trnV_GAC_104248_Cycas_debaoensis

AGGGATATAACTCAGCGGTAGAGTGTCACCTTGACGTGGTGGAAGtCATCAGTTCGAGCCTGATTATCCCTA

>trnQ_UUG_8078_Cycas_debaoensis

TGGGGCGTGGCCAAGCGGTAAGGCAGCGGGTTTTGGTCCTGTTATtCGGAGGTTCGAATCCTTCCGTCCCAG

>trnL_CAA_97914_Cycas_debaoensis

GCCTTGATGGTGGAATGGTaGACACGCGAGACTCAAAATCTCGTGCTAAAGAGCGTGGAGGTTCGAGTCCTCTTCAAGGCA

>trnR_ACG_111990_Cycas_debaoensis

GGGCCTGTAGCTCAGAGGAttAGAGCACGTGGCTACGAACCACGGTGtCGGGGGTTCGAATCCCTCCTCGCCCA

>trnI_CAU_88785_Cycas_debaoensis

GCATCCATGGCTGAACGGTtAAAGCGCCCAACTCATAATTGGcGAATtCGCAGGTTCAATTCCTGCTGGATGCA

>trnP_UGG_69803_Cycas_debaoensis

AGGGATGTAGCGCAGCTtGGTAGCGCGTTTGTTTTGGGTACAAAATGtCGCGGGTTCAAATCCTGTCATCCCTA

>trnR_UCU_11752_Cycas_debaoensis

GCGTCCATCGTCTAATGGAtAGGACAGGGGTCTTCTAAACCTCCGGTATAGGTTCAAATCCTATTGGACGTA

>trnR_CCG_59698_Cycas_debaoensis

GGGTTGGTAGCTCAGTGGAtcAGAGACCATGGTCCCGGACCATGAAGtCAAGGGTTCGAATCCCTCCTAGCCCA

>trnA_UGC_142660_Cycas_debaoensis

GGGGATATAGCTCAGTTGGTAGAGCTCCGCTCTTGCATGGCGGATGtCAGCGGTTCGAGTCCGCTTATCTCCA

>trnK_UUU_1716_Cycas_debaoensis

GGGTTGCTAACTCAATGGCAGAGTACTCGGCTTTTAACCGACGAGtTCCGGGTTCGAGTCCCGGGCAACCCA

>trnW_CCA_69559_Cycas_debaoensis

GCGCTCTTAGTTCAGTCcGGTAGAACGTAGGTCTCCAAAACCTAATGcCGTAGGTTCAAATCCTACAGAGCGTG

>trnP_GGG_118574_Cycas_debaoensis

CGGAGCATGGCGCAGCTtGGTAGCGTGCCATCTTGGGGTGATGGAGGtCGCAGGTTCAAATCCTGCTGCTCCGA

>trnC_GCA_28552_Cycas_debaoensis

GGCGACATAGCCAAGTGGTAAGGCAGAGGACTGCAAATCCTTTAtCCCCAGTTCAAATCCGGGTGTCGCCT

>trnS_GCU_9648_Cycas_debaoensis

GGAGAGATGGCTGAGCGGAccAAAGCGGTGGATTGCTAATCCATTGTACGAGCTAtTCGTACCGAGGGTTCGAATCCCTCTCTCTCCG

>trnM_CAU_55239_Cycas_debaoensis

ACCTACTTAACTCAGTGGTtAGAGTATCGCTTTCATACGGCGGGAGtCATTGGTTCAAATCCAATAGTAGGTA

>trnM_CAU_37229_Cycas_debaoensis

TGCGGGGTAGAGCAGTTtGGTAGCTCGCAAGGCTCATAACCTTGAGGtCACGGGTTCAAATCCCGTCTCCGCCA

>trnG_GCC_37035_Cycas_debaoensis

GCGGGTATGGTTGAGTGGTAAAATTTCTCCTTGCCAAGGAGAAGaTGCGGGTTCGATTCCCGCTACCCGCC

>trnV_UAC_54436_Cycas_debaoensis

AGGGCTATAGCTCAGCAaGGTAGAGCACCTCGTTTACACCGAGAAGGtCTACGGTTCGAGCCCGTATAGCCCTA

>trnH_GUG_161706_Cycas_debaoensis

GCGGACGTAGCCAAGTGGAtcAAGGCAGTGGATTGTGAATCCACCACgCGCGGGTTCAATCCCCGTCGTTCGCC

>trnM_CAU_41168_Ginkgo_biloba

TGCGGGGTAGAGCAGTTtGGTAGCTCGCAAGGCTCATAACCTTGAGGtCACGGGTTCAAATCCCGTCTCCGCCG

>trnS_UGA_40045_Ginkgo_biloba

GGAGAGATGGCCGAGTGGTtcATGGCTCCGGTCTTGAAAACCGGTATAGTTACAAAaAAACTATCGAGGGTTCGAACCCCTCTCTCTCCT

>trnD_GUC_34119_Ginkgo_biloba

GGGATTGTAGTTCAATCGGTtAGAGTACCGCCCTGTCAAGACGGAAGtTGCGGGTTCGAGCCCCGTCAGTCCCG

>trnT_UGU_50879_Ginkgo_biloba

GCCTGCTTAGCTCAGAGGTtAGAGCATCGCACTTGTAATGCGATGGtCATCGGTTCGATCCCGATAGTCGGCT

>trnS_GCU_9881_Ginkgo_biloba

GGAGAGATGGCTGAGCGGAccAAAGCGGCGAATTGCTAATTCGTTGTACGAGCTATCcGTACCGAGGGTTCGAATCCCTCTCTCTCCG

>trnM_CAU_57058_Ginkgo_biloba

ACCCACTTAACTCAGTGGTtAGAGTATCGCTTCCATACGGCGGGAGtCATTGGTTCAAATCCAATAGTAGGTA

>trnP_UGG_71928_Ginkgo_biloba

AGGGATGTAGCGCAGTTtGGTAGCGCGTTTGTTTTGGGTACAAAATGtCGCGGGTTCAAATCCTGTCATCCCTA

>trnG_UCC_34465_Ginkgo_biloba

GGGTCGATGCCCGAGCGGTtaATGAGGACGGACTGTAAATTCGTTGGCAATATGCCTaCGCTGGTTCAAATCCAGCTCGACCCA

>trnN_GUU_115753_Ginkgo_biloba

TCCTCAGTAGCTCAGTGGTAGAGCGGTCGGCTGTTAACTGACTGGtCGTAGGTTCAAATCCTACTTGGGGAG

>trnR_ACG_141225_Ginkgo_biloba

GGGCCTGTAGCTCAGAGGAttAGAGCACGTGGCTACGAACCACGGTGtCGGGGGTTCGAATCCCTCCTCGCCCA

>trnV_GAC_107181_Ginkgo_biloba

GGGGATATAACTCAGCGGTAGAGTGTCACCTTGACGTGGTGGAAGtCATCAGTTCGAGCCTGATTATCCCTA

>trnW_CCA_71691_Ginkgo_biloba

GCGCTCTTAGTTCAGTTcGGTAGAACGTAGGTCTCCAAAACCTGATGtCGTAGGTTCAAATCCTACAGAGCGTG

>trnQ_UUG_8211_Ginkgo_biloba

TGGGGCGTGGCCAAGTGGTAAGGCAGCAGGTTTTGGTCCTGTTACtCGGAGGTTCAAATCCTTCCGCCCCAG

>trnY_AUA_29738_Ginkgo_biloba

GGCGACATAGCTAAGTGGTAAAGCGGAGGACTATAAATCCTCGATCCtcGAtTCTCGGTTCAAATCCGGGgGTCGTCT

>trnL_CAA_100728_Ginkgo_biloba

GGGGATATGGCGGAATTGGTaGACGCTACGGACTCAAAATCCGTCGgctTTAtAgACCGTGAGGGTTCAAGTCCCTCTATTCCCA

>trnA_UGC_145059_Ginkgo_biloba

GGGGATATAGCTCAGTTGGTAGAGCTCCGCTCTTGCATGGCGGATGtCAGCGGTTCGAGTCCGCTTATCTCCA

>trnC_GCA_29928_Ginkgo_biloba

GGCGACATAGCCAAGTGGTAAGGCGGAGGACTGCAAATCCTCCAtCCCCGGTTCAAATCCGGGTGTCGCCT

>trnH_GUG_91360_Ginkgo_biloba

GCGGACGTAGCCAAGTGGAtcAAGGCAGTGGATTGTGAATCCACCACgCGTGGGTTCAATCCCCGTCGTTCGTC

>trnY_GUA_56265_Ginkgo_biloba

GGGGCTATAGCTCAGCAaGGTAGAGCACCTCGTTtataataagaacttcaatctgtTCTACCGAGAAGGtCTACGGTTCGAGCCCGTATAGCCCTA

>trnR_UCU_12046_Ginkgo_biloba

GCGTCCATCGTCTAATGGAtAGGACAGAGGTCTTCTAAACCTTCGGTATAGGTTCAAATCCTATTGGACGTA

>trnK_UUU_1941_Ginkgo_biloba

GGGTTGCTAACTCAATGGCAGAGTACTCGGCTTTTAACCGACGAGtTCCGGGTTCGAGTCCCGGGCAACCCA

>trnP_GGG_134743_Ginkgo_biloba

CGGAGCATGGCGCAACTtGGTAGCGTGCCATCGCGGGGTGATGGAGGtCGTGGGTTCAAATCCCATTGCTCCGA

>trnL_CAA_155435_Ginkgo_biloba

GCCTTGATGGTGAAATGGTaGACACGCGAGACTCAAAATCTCGTGCTAAAGAGCGTGGAGGTTCGAGTCCTCTTCAAGGCA

>trnF_GAA_52283_Ginkgo_biloba

GCCGGGATAGCTCAGTTGGTAGAGCAGAGGACTGAAAATCCTCGTGcCACCAGTTCAAATCTGGTTCCTGGCA

>trnR_CCG_61512_Ginkgo_biloba

GGGTTGGTAGCTCAGTGGAtcAGAGCCCATGGTTCCGGACCATGAAGtCAAGGGTTCAAATCCCTTCTAGCCCA

>trnH_GUG_24_Ginkgo_biloba

GCGGACGTAGCCAAGTGGAttAAGGCAGTGGATTGTGAATCCACCACgCGCGGGTTCAATCCCCGTCGTTCGCC

>trnE_UUC_34608_Ginkgo_biloba

GCCCCTATCGTCTAGTGGCccAGGACATCTCTCTTTCAAGGAGGCAGCGGGGATTCGACTTCCCCTAGGGGTA

>trnL_UAG_134523_Ginkgo_biloba

GCCGCCATGGTGAAATTGGTaGACACGCTGCTCTTAGGAAGCAGTGCTAgAGcaTCTCGGTTCGAGTCCGAGTGGCGGCA

>trnG_GCC_40978_Ginkgo_biloba

GCGGGTATGGTTTAATGGTAAAATTTCTCTTTGCCAGGGAGAAGaTGCGGGTTCGATCCCCGCTACCCGCC

>trnS_GGA_10910_Ginkgo_biloba

GCGGGTATAGTTTAGTGGTAaaagtgtgatttcgactatAACCCCTAGCCTTCCAAGCTAACGaTGCGGGTTCGATTCCCGCTACCCGCT

>trnT_GGU_35960_Ginkgo_biloba

GCCCTTTTAACTCAGTGGTAGAGTAACGCCATGGTAAGGCGTAAGtCATCGGTTCAAATCCGATAAAGGGCT

>trnC_ACA_29654_Ginkgo_biloba

GGCGAGATAGCCAAGTGGTAAGGCAGAGGACTACAAATCCTTGATcCCCGGGTTCCAACCCGGggaTCGCCT

>trnI_CAU_90908_Ginkgo_biloba

GCATCCATGGCTGAATGGTtAAAGCGCCCAACTCATAATTGGcGAATtCGCGGGTTCAATTCCTGCTGGATGCA

>trnS_GCU_9377_Dioon_spinulosum

GGAGAGATGGCTGAGCGGCccAAAGCGGCGGATTGCTAATCCGTTGTACGAACTATTCGTACCGAGGGTTCGAATCCCTCTCTCTCCG

>trnK_UUU_1610_Dioon_spinulosum

GGGTTGCTAACTCAATGGTAGAGTACTCGGCTTTTAACCGACGAGtTCCGGGTTCGAGTCCCGGGCAACCCA

>trnP_GGG_118622_Dioon_spinulosum

CGGGGCATGGCGTAGCTtGGTAGCGTGCCATCTTGGGGTGGTGGAGGtCGTGGGTTCAAATCCCGCTGCTCCGA

>trnC_GCA_28269_Dioon_spinulosum

GGCGGCATAGCCAAGTGGTAAGGCAGAGGACTGCAAATCCTTTAtCCCCAGTTCAAATCCGGGTGTCGCCT

>trnR_ACG_118622_Dioon_spinulosum

GGGCCTGTAGCTCAGAGGAttAGAGCACGTGGCTACGAACCACGGTGtCGGGGGTTCGAATCCCTCCTCGCCCA

>trnT_UGU_48239_Dioon_spinulosum

GCCCGCTTAGCTCAGAGGTtAGAGCATCGCACTTGTAATGCGATGGtCATCGGTTCGATTCCGATAGCCGGCT

>trnS_GGA_47009_Dioon_spinulosum

GGAAAGATGGCCGAGCGGTtcAAGGCGTAGCATTGGAACTGCTATGTAGGCTTTTGTTTACCGAGGGTTCGAATCCCTCTCTTTCCG

>trnH_GUG_161222_Dioon_spinulosum

GCGGACGTAGCCAAGTGGAtcAAGGCAGTGGATTGTGAATCCACCACgCGCGGGTTCAATCCCCGTCGTTCGCC

>trnM_CAU_56165_Dioon_spinulosum

ACCTACTTAACTCAGTGGTtAGAGTATCGCTTTCATACGGCGGGAGtCATTGGTTCAAATCCAATAGTAGGTA

>trnL_UAG_118837_Dioon_spinulosum

GCCGCCATGGTGAAATTGGTaGACACGCTGCTCTTAGGAAGCAGTGCTAgAGcaTCTCGGTTCGAATCCGAGTGGCGGCA

>trnS_UGA_37484_Dioon_spinulosum

GGAGAGATGGCCGAGTGGTtcATGGCTCCGGTCTTGAAAACCGGTATAGTTACAAAAAGCTATCGAGGGTTCGAATCCCTCTCTCTCCT

>trnV_UAC_55383_Dioon_spinulosum

AGGGCTATAGCTCAGCAaGGTAGAGCACCTCGTTTACACCGAGAAGGtCTACGGTTCGAGCCCGTATAGCCCTA

>trnD_GUC_31761_Dioon_spinulosum

GGGATTGTAGTTCAATTGGTcAGAGTACCGTCCTGTCAAGACGGAAGtTACGGGTTCGAGCCCCGTCAGTCCCG

>trnL_UAA_48715_Dioon_spinulosum

GGGGATATGGCGGAATTGGTaGACGCTACGGACTTAAAATCCGTCGgctTTAtAgACCGTGAGAGTTCAAATCTCTCTATCCCCA

>trnA_UGC_107167_Dioon_spinulosum

GGGGATATAGCTCAGTTGGTAGAGCTCCGCTCTTGCATGGCGGATGtCAGCGGTTCGAGTCCGCTTATCTCCA

>trnV_GAC_146145_Dioon_spinulosum

AGGGATATAACTCAGCGGTAGAGTGTCACCTTGACGTGGTGGAAGtCATCAGTTCGAGCCTGATTATCCCTA

>trnT_GGU_33411_Dioon_spinulosum

GCCCTTTTAGCTCGGCGGTATAGTAACGCCATGGTAAGGCATAAGtCATCGGTTCAAATCTGATAAAGGGTT

>trnE_UUC_143118_Dioon_spinulosum

GGGCTATTAGCTCAGTGGTAGAGCGCGCCCCTTTCACGGGCGAGGtCTCTGGTTCAAGCCCAGGATAGCCCA

>trnM_CAU_38618_Dioon_spinulosum

TGCGGGGTAGAGCAGTTtGGTAGCTCGCAAGGCTCATAACCTTGAGGtCACGGGTTCAAATCCCGTCTCCGCCG

>trnE_UUC_32282_Dioon_spinulosum

GCCCCTATCGTCTAGTGGCccAGGACATCTCTCTTTCAAGGAGGCAGCGGGGATTCGACTTCCCCTAGGGGTA

>trnI_CAU_88511_Dioon_spinulosum

GCATCCATGGCTGAACGGTtAAAGCGCCCAACTCATAATTGGcGAATtCGCAGGTTCAATTCCTGCTGGATGCA

>trnG_GCC_38421_Dioon_spinulosum

GCGGGTATGGTTGAGTGGTAAAATTTCTCCTTGCCAGGGAGAAGaTGCGGGTTCGATCCCCGCTACCCGCC

>trnQ_UUG_7818_Dioon_spinulosum

TGGGGCGTGGCCAAGCGGTAAGGCAGCGGGTTTTGGTCCTGTTACtCGGAGGTTCGAATCCTTCCGTCCCAG

>trnR_CCG_60617_Dioon_spinulosum

GGGTTAGTAGCTCAGTGGAtcAGAGCCCATGGTCCCGGACCATGAAGtCAAGGGTTCAAATCCCTTCTAGCCTA

>trnR_UCU_11473_Dioon_spinulosum

GCGTCCATCGTCTAATGGAtAGGACAGAGGTCTTCTAAACCTCTGGTATAGGTTCAAATCCTATTGGACGTA

>trnF_GAA_49706_Dioon_spinulosum

GCCGGGATAGCTCAGTTGGTAGAGCAGAGGACTGAAAATCCTCGTGcCACCAGTTCAAATCTGGTTCCTGGCA

>trnP_UGG_69973_Dioon_spinulosum

AGGGATGTAGCGCAGCTtGGTAGCGCGTTTGTTTTGGGTACAAAATGtCGCGGGTTCAAATCCTGTCATCCCTA

>trnN_GUU_137554_Dioon_spinulosum

TCCTCAGTAGCTCAGCGGTAGAGCGGTCGGCTGTTAACTGACTGGtCGTAGGTTCAAATCCTACTTGGGGAG

>trnW_CCA_69732_Dioon_spinulosum

GCGCTCTTAGTTTAGTCcGGTAGAACGTAGGTCTCCAAAACCTAATGcCGTAGGTTCAAATCCTACAGAGCGTG

>trnY_GUA_32134_Dioon_spinulosum

GGGTCGATGCCCGAGTGGTtaATGAGGACGGACTGTAAATTCGTTGGCAATATGCCTaCGCTGGTTCAAATCCAGCTCGACCCA

>trnL_CAA_97662_Dioon_spinulosum

GCCTTGATGGTGGAATGGTaGACACACGAGACTCAAAATCTCGTGCTAAAGAGCGTGGAGGTTCGAGTCCTCTTCAAGGCA
