# Supplementary figures and images for: Evolutionary analysis of chloroplast tRNA of Gymnosperm revealed the novel structural variation and evolutionary aspect
Source: PeerJ. 2020 Nov 25;8:e10312. doi: 10.7717/peerj.10312 (PMC7698693; doi:10.7717/peerj.10312)

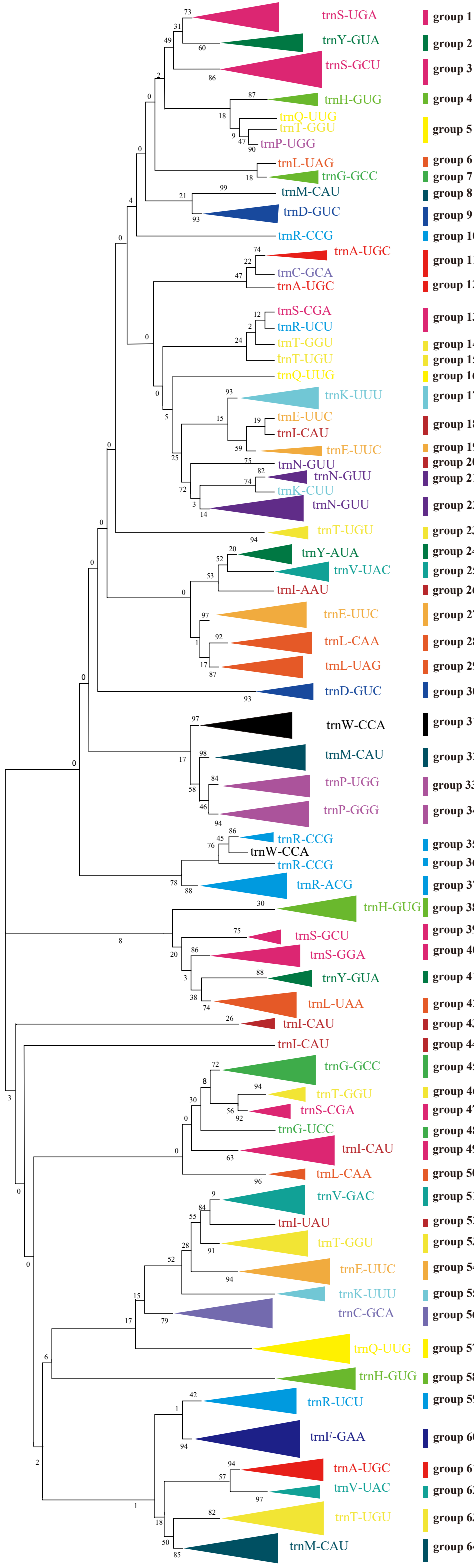

Supplement: Supplemental Information 2 — Multiply tRNAs are shown by different colors. Different groups are marked by different strings. The phylogenetic clades with low bootstrap replicates were collapsed with 50% cutoff values. Phylogenetic analysis illustrates that Gymnosperm chloroplast tRNA derived from common multiple ancestors. [file peerj-08-10312-s002.pdf]

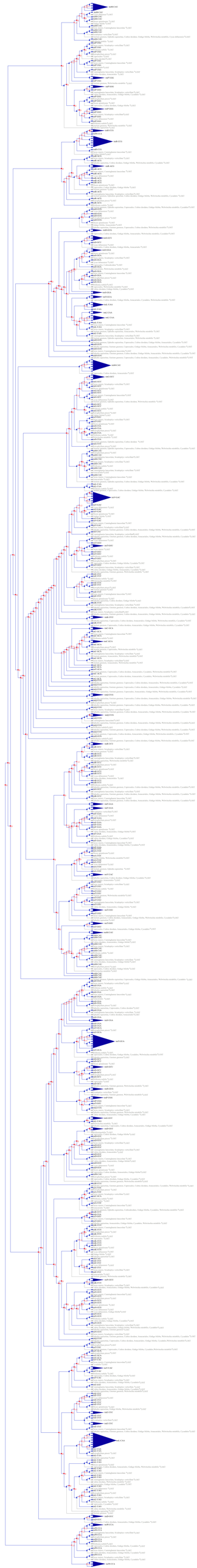

Supplement: Supplemental Information 3 — 153 duplication events (duplication and conditional duplication) are detected in all of the gymnosperm chloroplast tRNA genes, and gene loss events are detected with 220. Blue: Duplication events; Gray: Loss events; D: Duplication node; cD: Conditional Duplication node. [file peerj-08-10312-s003.pdf]
