# Supplementary material for: Evolutionary analysis of chloroplast tRNA of Gymnosperm revealed the novel structural variation and evolutionary aspect
Source: PeerJ. 2020 Nov 25;8:e10312. doi: 10.7717/peerj.10312 (PMC7698693; doi:10.7717/peerj.10312)
Supplement: Supplemental Information 5 — The acceptor arm of chloroplast tRNAs contains 3 bp to 7 bp, where 357 have 7 bp, 13 have 6 bp, and the remaining tRNAs contain no more than 5 bp. The anticodon arms of chloroplast tRNAs mainly contain 5 bp. The anticodon loop of gymnosperm chloroplast tRNAs generally contains 7 nt, and thus the sequence of the anticodon loop is typically conserved. [file peerj-08-10312-s005.docx]

| Table S1 Nucleotide composition in different parts of clover-structure of chloroplast genome tRNA. | | | | | | | | |
| --- | --- | --- | --- | --- | --- | --- | --- | --- |
|  | **AC-arm** | **D-arm** | **D-loop** | **ANC-arm** | **ANC-loop** | **Variable region** | **Ψ-arm** | **Ψ-loop** |
| **Alanine** | | | | | | | | |
| Welwitschia_mirabilis_82685 | 7 | 4 | 8 | 5 | 7 | 5 | 7 | 8 |
| Cycas_debaoensis_142660 | 7 | 4 | 8 | 5 | 7 | 5 | 7 | 8 |
| Gnetum_gnemon_34443 | 7 | 4 | 8 | 5 | 7 | 5 | 7 | 8 |
| Ephedra_equisetina_96165 | 7 | 4 | 8 | 5 | 7 | 5 | 7 | 8 |
| Cedrus_deodara_36537 | 7 | 4 | 8 | 5 | 7 | 5 | 7 | 8 |
| Ginkgo_biloba_145059 | 7 | 4 | 8 | 5 | 7 | 5 | 7 | 8 |
| Cunninghamia_lanceolata_50692 | 7 | 4 | 8 | 5 | 7 | 5 | 7 | 8 |
| Wollemia_nobilis_41240 | 7 | 4 | 8 | 5 | 7 | 5 | 7 | 8 |
| Dioon_spinulosum_107167 | 7 | 4 | 8 | 5 | 7 | 5 | 7 | 8 |
| Sciadopitys_verticillata_12642 | 7 | 4 | 8 | 5 | 7 | 5 | 7 | 8 |
| **Arginine** | | | | | | | | |
| Welwitschia_mirabilis_87261 | 7 | 4 | 9 | 5 | 7 | 5 | 5 | 7 |
| Taxus_mairei_45150 | 7 | 4 | 9 | 5 | 7 | 5 | 5 | 7 |
| Retrophyllum_piresii_112715 | 7 | 4 | 9 | 5 | 7 | 5 | 5 | 7 |
| Cycas_debaoensis_111990 | 7 | 4 | 9 | 5 | 7 | 5 | 5 | 7 |
| Gnetum_gnemon_19249 | 7 | 4 | 9 | 5 | 7 | 5 | 5 | 7 |
| Ephedra_equisetina_77681 | 7 | 4 | 9 | 5 | 7 | 5 | 5 | 7 |
| Cedrus_deodara_41120 | 7 | 4 | 9 | 5 | 7 | 5 | 5 | 7 |
| Ginkgo_biloba_141225 | 7 | 4 | 9 | 5 | 7 | 5 | 5 | 7 |
| Cunninghamia_lanceolata_46913 | 7 | 4 | 9 | 5 | 7 | 5 | 5 | 7 |
| Wollemia_nobilis_46496 | 7 | 4 | 9 | 5 | 7 | 5 | 5 | 7 |
| Dioon_spinulosum_118622 | 7 | 4 | 9 | 5 | 7 | 5 | 5 | 7 |
| Sciadopitys_verticillata_8524 | 7 | 4 | 9 | 5 | 7 | 5 | 5 | 7 |
| Retrophyllum_piresii_25821 | 7 | 4 | 9 | 5 | 7 | 5 | 5 | 7 |
| Cycas_debaoensis_59698 | 7 | 3 | 9 | 5 | 7 | 6 | 4 | 7 |
| Gnetum_gnemon_72128 | 7 | 4 | 9 | 5 | 7 | 5 | 5 | 7 |
| Cedrus_deodara_106734 | 7 | 4 | 9 | 5 | 7 | 5 | 5 | 7 |
| Ginkgo_biloba_61512 | 7 | 4 | 9 | 5 | 7 | 5 | 5 | 7 |
| Wollemia_nobilis_103032 | 7 | 4 | 9 | 5 | 7 | 7 | 5 | 7 |
| Dioon_spinulosum_60617 | 7 | 4 | 9 | 5 | 7 | 5 | 5 | 7 |
| Sciadopitys_verticillata_90492 | 7 | 4 | 9 | 5 | 7 | 5 | 5 | 7 |
| Welwitschia_mirabilis_44496 | 7 | 4 | 9 | 4 | 7 | 6 | 5 | 7 |
| Welwitschia_mirabilis_48561 | 7 | 4 | 9 | 5 | 7 | 5 | 5 | 7 |
| Welwitschia_mirabilis_34644 | 7 | 4 | 8 | 5 | 7 | 4 | 5 | 7 |
| Taxus_mairei_81144 | 7 | 4 | 8 | 5 | 7 | 4 | 5 | 7 |
| Retrophyllum_piresii_114287 | 7 | 4 | 8 | 5 | 7 | 4 | 5 | 7 |
| Cycas_debaoensis_11752 | 7 | 4 | 8 | 5 | 7 | 4 | 5 | 7 |
| Gnetum_gnemon_81775 | 7 | 4 | 8 | 5 | 7 | 4 | 5 | 7 |
| Ephedra_equisetina_32616 | 7 | 4 | 8 | 5 | 7 | 4 | 5 | 7 |
| Ginkgo_biloba_12046 | 7 | 4 | 8 | 5 | 7 | 4 | 5 | 7 |
| Cunninghamia_lanceolata_42688 | 7 | 4 | 8 | 5 | 7 | 4 | 5 | 7 |
| Wollemia_nobilis_6259 | 7 | 4 | 8 | 5 | 7 | 4 | 5 | 7 |
| Dioon_spinulosum_11473 | 7 | 4 | 8 | 5 | 7 | 4 | 5 | 7 |
| Sciadopitys_verticillata_90759 | 7 | 4 | 8 | 5 | 7 | 4 | 5 | 7 |
| Cedrus_deodara_51513 | 7 | 4 | 8 | 5 | 7 | 4 | 5 | 7 |
| **Asparagine** | | | | | | | | |
| Welwitschia_mirabilis_100730 | 7 | 4 | 7 | 4 | 7 | 6 | 5 | 7 |
| Taxus_mairei_45672 | 7 | 4 | 7 | 5 | 7 | 5 | 5 | 7 |
| Retrophyllum_piresii_76577 | 7 | 4 | 7 | 5 | 7 | 5 | 5 | 7 |
| Cycas_debaoensis_138071 | 7 | 4 | 7 | 5 | 7 | 5 | 5 | 7 |
| Gnetum_gnemon_19530 | 7 | 4 | 7 | 5 | 7 | 7 | 5 | 7 |
| Ephedra_equisetina_77973 | 7 | 4 | 7 | 5 | 7 | 7 | 5 | 7 |
| Cedrus_deodara_41931 | 7 | 4 | 7 | 5 | 7 | 5 | 5 | 7 |
| Ginkgo_biloba_115753 | 7 | 4 | 7 | 5 | 7 | 5 | 5 | 7 |
| Cunninghamia_lanceolata_46290 | 7 | 4 | 7 | 5 | 7 | 5 | 5 | 7 |
| Wollemia_nobilis_47307 | 7 | 4 | 7 | 5 | 7 | 5 | 5 | 7 |
| Dioon_spinulosum_137554 | 7 | 4 | 7 | 5 | 7 | 5 | 5 | 7 |
| Sciadopitys_verticillata_8201 | 7 | 4 | 7 | 5 | 7 | 5 | 5 | 7 |
| **Asparagicacid** | | | | | | | | |
| Welwitschia_mirabilis_71649 | 7 | 4 | 9 | 5 | 7 | 5 | 5 | 7 |
| Taxus_mairei_99499 | 7 | 4 | 9 | 5 | 7 | 5 | 5 | 7 |
| Cycas_debaoensis_31566 | 7 | 4 | 9 | 5 | 7 | 5 | 5 | 7 |
| Gnetum_gnemon_78589 | 7 | 4 | 9 | 5 | 7 | 5 | 5 | 7 |
| Ephedra_equisetina_34603 | 7 | 4 | 9 | 5 | 7 | 5 | 5 | 7 |
| Cedrus_deodara_98431 | 7 | 4 | 9 | 5 | 7 | 5 | 5 | 7 |
| Ginkgo_biloba_34119 | 7 | 4 | 9 | 5 | 7 | 5 | 5 | 7 |
| Cunninghamia_lanceolata_23222_ | 7 | 4 | 9 | 5 | 7 | 5 | 5 | 7 |
| Wollemia_nobilis_71649 | 7 | 4 | 9 | 5 | 7 | 5 | 5 | 7 |
| Dioon_spinulosum_31761 | 7 | 4 | 9 | 5 | 7 | 5 | 5 | 7 |
| Sciadopitys_verticillata_64130 | 7 | 4 | 9 | 5 | 7 | 5 | 5 | 7 |
| Retrophyllum_piresii_25574 | 7 | 4 | 9 | 5 | 7 | 5 | 5 | 7 |
| Retrophyllum_piresii_133138 | 7 | 4 | 9 | 5 | 7 | 5 | 5 | 7 |
| **Cysteine** | | | | | | | | |
| Ginkgo_biloba_29654 | 7 | 3 | 9 | 5 | 7 | 5 | 5 | 6 |
| Welwitschia_mirabilis_34644 | 7 | 3 | 9 | 5 | 7 | 4 | 5 | 7 |
| Taxus_mairei_97135 | 7 | 3 | 9 | 5 | 7 | 4 | 5 | 7 |
| Cycas_debaoensis_28552 | 7 | 3 | 9 | 5 | 7 | 4 | 5 | 7 |
| Gnetum_gnemon_97818 | 7 | 3 | 9 | 5 | 7 | 5 | 5 | 7 |
| Ephedra_equisetina_17629 | 7 | 3 | 9 | 5 | 7 | 5 | 5 | 7 |
| Cedrus_deodara_96061 | 7 | 3 | 9 | 5 | 7 | 4 | 5 | 7 |
| Ginkgo_biloba_29928 | 7 | 3 | 9 | 5 | 7 | 4 | 5 | 7 |
| Cunninghamia_lanceolata_26571 | 7 | 3 | 9 | 5 | 7 | 4 | 5 | 7 |
| Wollemia_nobilis_134757 | 7 | 3 | 9 | 5 | 7 | 4 | 5 | 7 |
| Dioon_spinulosum_28269 | 7 | 3 | 9 | 5 | 7 | 4 | 5 | 7 |
| Sciadopitys_verticillata_137519 | 7 | 3 | 9 | 4 | 7 | 5 | 5 | 7 |
| Retrophyllum_piresii_108018 | 7 | 4 | 8 | 4 | 11 | 23 | 5 | 7 |
| **Glutamine** | | | | | | | | |
| Welwitschia_mirabilis_37177 | 7 | 3 | 9 | 5 | 7 | 5 | 5 | 7 |
| Taxus_mairei_78840 | 7 | 3 | 9 | 5 | 7 | 5 | 5 | 7 |
| Retrophyllum_piresii_79005 | 7 | 3 | 9 | 5 | 7 | 6 | 5 | 7 |
| Cycas_debaoensis_8078 | 7 | 3 | 9 | 5 | 7 | 5 | 5 | 7 |
| Gnetum_gnemon_79080 | 7 | 3 | 9 | 5 | 7 | 6 | 5 | 7 |
| Ephedra_equisetina_34120 | 7 | 3 | 9 | 5 | 7 | 6 | 5 | 7 |
| Cedrus_deodara_76449 | 7 | 3 | 9 | 5 | 7 | 5 | 5 | 7 |
| Ginkgo_biloba_8211 | 7 | 3 | 9 | 5 | 7 | 5 | 5 | 7 |
| Wollemia_nobilis_9806 | 7 | 3 | 9 | 5 | 7 | 6 | 5 | 7 |
| Dioon_spinulosum_7818 | 7 | 3 | 9 | 5 | 7 | 5 | 5 | 7 |
| Cunninghamia_lanceolata_7625 | 7 | 3 | 12 | 5 | 7 | 6 | 5 | 7 |
| Sciadopitys_verticillata_94297 | 7 | 3 | 9 | 5 | 7 | 6 | 5 | 7 |
| Cunninghamia_lanceolata_46041 | 7 | 3 | 9 | 5 | 7 | 6 | 5 | 7 |
| Sciadopitys_verticillata_94370 | 7 | 3 | 9 | 5 | 7 | 6 | 5 | 7 |
| **Glutamicacid** | | | | | | | | |
| Taxus_mairei_99898 | 7 | 4 | 9 | 5 | 7 | 4 | 5 | 7 |
| Ginkgo_biloba_34608 | 7 | 4 | 9 | 5 | 7 | 4 | 5 | 7 |
| Welwitschia_mirabilis_37389 | 7 | 4 | 9 | 5 | 7 | 4 | 5 | 7 |
| Retrophyllum_piresii_306 | 7 | 4 | 9 | 5 | 7 | 4 | 5 | 7 |
| Cycas_debaoensis_32087 | 7 | 4 | 9 | 5 | 7 | 4 | 5 | 7 |
| Ephedra_equisetina_34302 | 7 | 4 | 9 | 5 | 7 | 4 | 5 | 7 |
| Cunninghamia_lanceolata_22791 | 7 | 4 | 9 | 5 | 7 | 4 | 5 | 7 |
| Dioon_spinulosum_32282 | 7 | 4 | 9 | 5 | 7 | 4 | 5 | 7 |
| Gnetum_gnemon_35348 | 7 | 4 | 7 | 5 | 7 | 5 | 5 | 7 |
| Cedrus_deodara_35405 | 7 | 4 | 7 | 5 | 7 | 5 | 5 | 7 |
| Wollemia_nobilis_130551 | 7 | 4 | 7 | 5 | 7 | 5 | 5 | 7 |
| Sciadopitys_verticillata_13581 | 7 | 4 | 7 | 5 | 7 | 5 | 5 | 7 |
| Welwitschia_mirabilis_105669 | 7 | 4 | 7 | 5 | 7 | 5 | 5 | 7 |
| Retrophyllum_piresii_106909 | 7 | 4 | 7 | 5 | 7 | 5 | 5 | 7 |
| Cycas_debaoensis_143585 | 7 | 4 | 7 | 5 | 7 | 5 | 5 | 7 |
| Gnetum_gnemon_78878 | 7 | 4 | 9 | 5 | 7 | 4 | 5 | 7 |
| Ephedra_equisetina_97046 | 7 | 4 | 7 | 5 | 7 | 11 | 5 | 7 |
| Cedrus_deodara_98926 | 7 | 4 | 9 | 5 | 7 | 4 | 5 | 7 |
| Cunninghamia_lanceolata_51599 | 7 | 4 | 7 | 5 | 7 | 5 | 5 | 7 |
| Wollemia_nobilis_40148 | 7 | 4 | 9 | 5 | 7 | 4 | 5 | 7 |
| Dioon_spinulosum_143118 | 7 | 4 | 7 | 5 | 7 | 5 | 5 | 7 |
| Sciadopitys_verticillata_64546 | 7 | 4 | 9 | 5 | 7 | 4 | 5 | 7 |
| **Glycine** | | | | | | | | |
| Welwitschia_mirabilis_9009 | 7 | 3 | 9 | 5 | 7 | 4 | 5 | 7 |
| Taxus_mairei_105489 | 7 | 4 | 8 | 5 | 7 | 4 | 5 | 7 |
| Retrophyllum_piresii_5971 | 7 | 4 | 7 | 5 | 7 | 4 | 5 | 7 |
| Cycas_debaoensis_37035 | 7 | 3 | 9 | 5 | 7 | 4 | 5 | 7 |
| Gnetum_gnemon_107307 | 7 | 3 | 9 | 5 | 7 | 4 | 5 | 7 |
| Ephedra_equisetina_8708 | 7 | 3 | 9 | 5 | 7 | 4 | 5 | 7 |
| Cedrus_deodara_28238 | 7 | 3 | 9 | 5 | 7 | 4 | 5 | 7 |
| Ginkgo_biloba_40978 | 7 | 4 | 7 | 5 | 7 | 4 | 5 | 7 |
| Gnetum_gnemon_111027 | 7 | 4 | 7 | 5 | 7 | 4 | 5 | 7 |
| Welwitschia_mirabilis_22487 | 7 | 4 | 7 | 5 | 7 | 5 | 5 | 7 |
| Retrophyllum_piresii_130291 | 7 | 3 | 9 | 5 | 7 | 4 | 5 | 7 |
| Cedrus_deodara_98774 | 7 | 4 | 9 | 5 | 7 | 4 | 5 | 7 |
| Ginkgo_biloba_34465 | 7 | 4 | 9 | 5 | 7 | 4 | 5 | 7 |
| Cunninghamia_lanceolata_17651 | 7 | 3 | 9 | 5 | 7 | 4 | 5 | 7 |
| Wollemia_nobilis_124623 | 7 | 3 | 9 | 5 | 7 | 4 | 5 | 7 |
| Dioon_spinulosum_38421 | 7 | 3 | 9 | 5 | 7 | 4 | 5 | 7 |
| Sciadopitys_verticillata_70419 | 7 | 4 | 7 | 5 | 7 | 4 | 5 | 7 |
| Wollemia_nobilis_90178 | 7 | 0 | 16 | 5 | 7 | 4 | 5 | 7 |
| **Histidine** | | | | | | | | |
| Welwitschia_mirabilis_118920 | 7 | 3 | 11 | 5 | 7 | 5 | 5 | 7 |
| Taxus_mairei_71194 | 7 | 3 | 12 | 5 | 7 | 5 | 5 | 7 |
| Retrophyllum_piresii_86732 | 7 | 3 | 10 | 5 | 7 | 5 | 5 | 7 |
| Cycas_debaoensis_161706 | 7 | 3 | 11 | 5 | 7 | 5 | 5 | 7 |
| Gnetum_gnemon_1622 | 7 | 3 | 11 | 5 | 7 | 5 | 5 | 7 |
| Ephedra_equisetina_61119 | 7 | 3 | 11 | 5 | 7 | 5 | 5 | 7 |
| Cedrus_deodara_69151 | 7 | 3 | 11 | 5 | 7 | 5 | 5 | 7 |
| Cunninghamia_lanceolata_134766 | 7 | 3 | 12 | 5 | 7 | 5 | 5 | 7 |
| Wollemia_nobilis_18545 | 7 | 3 | 11 | 5 | 7 | 5 | 5 | 7 |
| Dioon_spinulosum_161222 | 7 | 3 | 11 | 5 | 7 | 5 | 5 | 7 |
| Sciadopitys_verticillata_120805 | 7 | 3 | 11 | 5 | 7 | 5 | 5 | 7 |
| Ginkgo_biloba_24 | 7 | 3 | 11 | 5 | 7 | 5 | 5 | 7 |
| Ginkgo_biloba_91360 | 7 | 3 | 11 | 5 | 7 | 5 | 5 | 7 |
| **Isoleucine** | | | | | | | | |
| Taxus_mairei_119109 | 7 | 4 | 9 | 3 | 27 | 6 | 5 | 7 |
| Welwitschia_mirabilis_68903 | 7 | 3 | 10 | 5 | 7 | 5 | 5 | 7 |
| Wollemia_nobilis_21954 | 7 | 3 | 10 | 5 | 7 | 5 | 5 | 7 |
| Cunninghamia_lanceolata_60811 | 7 | 3 | 10 | 5 | 7 | 5 | 5 | 7 |
| Taxus_mairei_71583 | 7 | 3 | 10 | 5 | 7 | 5 | 5 | 7 |
| Taxus_mairei_23714 | 7 | 3 | 10 | 5 | 7 | 5 | 5 | 7 |
| Sciadopitys_verticillata_34364 | 7 | 3 | 10 | 5 | 7 | 5 | 5 | 7 |
| Retrophyllum_piresii_90145 | 7 | 3 | 10 | 5 | 7 | 5 | 5 | 7 |
| Ephedra_equisetina_60791 | 7 | 3 | 10 | 5 | 7 | 5 | 5 | 7 |
| Gnetum_gnemon_1264 | 7 | 3 | 10 | 5 | 7 | 5 | 5 | 7 |
| Cedrus_deodara_15398 | 7 | 3 | 10 | 5 | 7 | 5 | 5 | 7 |
| Cycas_debaoensis_88785 | 7 | 3 | 10 | 5 | 7 | 5 | 5 | 7 |
| Ginkgo_biloba_90908 | 7 | 3 | 10 | 5 | 7 | 5 | 5 | 7 |
| Dioon_spinulosum_88511 | 7 | 3 | 10 | 5 | 7 | 5 | 5 | 7 |
| Taxus_mairei_37361 | 7 | 4 | 7 | 5 | 7 | 5 | 5 | 7 |
| **Leucine** | | | | | | | | |
| Welwitschia_mirabilis_76801 | 7 | 3 | 10 | 5 | 7 | 13 | 5 | 7 |
| Taxus_mairei_chloroplast_31982 | 7 | 3 | 10 | 5 | 7 | 13 | 5 | 7 |
| Retrophyllum_piresii_97776 | 7 | 3 | 10 | 5 | 7 | 18 | 5 | 7 |
| Cycas_debaoensis_97914 | 7 | 3 | 10 | 5 | 7 | 13 | 5 | 7 |
| Gnetum_gnemon_8867 | 7 | 3 | 10 | 5 | 7 | 13 | 5 | 5 |
| Ephedra_equisetina_101987 | 7 | 3 | 10 | 5 | 7 | 12 | 5 | 7 |
| Cedrus_deodara_61986 | 7 | 3 | 10 | 5 | 7 | 13 | 5 | 7 |
| Cunninghamia_lanceolata_69095 | 7 | 3 | 10 | 5 | 7 | 13 | 5 | 7 |
| Wollemia_nobilis_30036 | 7 | 3 | 10 | 5 | 7 | 13 | 5 | 7 |
| Dioon_spinulosum_97662 | 7 | 3 | 10 | 5 | 7 | 13 | 5 | 7 |
| Sciadopitys_verticillata_23855 | 7 | 3 | 10 | 5 | 7 | 13 | 5 | 7 |
| Ginkgo_biloba_100728 | 7 | 3 | 11 | 5 | 7 | 16 | 5 | 7 |
| Ginkgo_155435 | 7 | 3 | 10 | 5 | 7 | 13 | 5 | 7 |
| Welwitschia_mirabilis_38388 | 7 | 3 | 11 | 5 | 7 | 16 | 5 | 7 |
| Taxus_mairei_115126 | 7 | 3 | 11 | 5 | 7 | 16 | 5 | 7 |
| Retrophyllum_piresii_16365 | 7 | 3 | 11 | 5 | 7 | 17 | 5 | 7 |
| Cycas_debaoensis_47471 | 7 | 3 | 11 | 5 | 7 | 16 | 5 | 7 |
| Gnetum_gnemon_77723 | 7 | 3 | 11 | 5 | 7 | 16 | 5 | 7 |
| Ephedra_equisetina_35050 | 7 | 3 | 11 | 5 | 7 | 16 | 5 | 7 |
| Cedrus_deodara_17278 | 7 | 3 | 11 | 5 | 7 | 16 | 5 | 7 |
| Cunninghamia_lanceolata_117276 | 7 | 3 | 11 | 5 | 7 | 16 | 5 | 7 |
| Wollemia_nobilis_113548 | 7 | 3 | 11 | 5 | 7 | 18 | 5 | 7 |
| Dioon_spinulosum_48715 | 7 | 3 | 11 | 5 | 7 | 16 | 5 | 7 |
| Sciadopitys_verticillata_80565 | 7 | 3 | 11 | 5 | 7 | 16 | 5 | 7 |
| Welwitschia_mirabilis_90133 | 7 | 3 | 11 | 5 | 7 | 11 | 5 | 7 |
| Taxus_mairei_50656 | 7 | 3 | 11 | 5 | 7 | 11 | 5 | 7 |
| Retrophyllum_piresii_72106 | 7 | 3 | 11 | 5 | 7 | 11 | 5 | 7 |
| Cycas_debaoensis_118790 | 7 | 3 | 11 | 5 | 7 | 11 | 5 | 7 |
| Gnetum_gnemon_27863 | 7 | 3 | 11 | 5 | 7 | 11 | 5 | 7 |
| Ephedra_equisetina_82595 | 7 | 3 | 11 | 5 | 7 | 3 | 5 | 7 |
| Cedrus_deodara_54755 | 7 | 3 | 11 | 5 | 7 | 11 | 5 | 7 |
| Ginkgo_biloba_134523 | 7 | 3 | 11 | 5 | 7 | 11 | 5 | 7 |
| Cunninghamia_lanceolata_103246 | 7 | 3 | 11 | 5 | 7 | 11 | 5 | 7 |
| Wollemia_nobilis_53167 | 7 | 3 | 11 | 5 | 7 | 11 | 5 | 7 |
| Dioon_spinulosum_118837 | 7 | 3 | 11 | 5 | 7 | 11 | 5 | 7 |
| Sciadopitys_verticillata_117202 | 7 | 3 | 11 | 5 | 7 | 11 | 5 | 7 |
| **Lysine** | | | | | | | | |
| Cunninghamia_lanceolata_46383 | 7 | 4 | 7 | 5 | 7 | 8 | 5 | 7 |
| Welwitschia_mirabilis_1145 | 7 | 4 | 7 | 4 | 7 | 6 | 5 | 7 |
| Taxus_mairei_73018 | 7 | 4 | 7 | 4 | 7 | 6 | 5 | 7 |
| Retrophyllum_piresii_82037 | 7 | 4 | 7 | 4 | 7 | 6 | 5 | 7 |
| Cycas_debaoensis_1716 | 7 | 4 | 7 | 4 | 7 | 6 | 5 | 7 |
| Gnetum_gnemon_112476 | 7 | 4 | 7 | 4 | 7 | 6 | 5 | 7 |
| Ephedra_equisetina_1209 | 7 | 4 | 7 | 4 | 7 | 6 | 5 | 7 |
| Cedrus_deodara_71308 | 7 | 4 | 7 | 4 | 7 | 6 | 5 | 7 |
| Ginkgo_biloba_1941 | 7 | 4 | 7 | 4 | 7 | 6 | 5 | 7 |
| Cunninghamia_lanceolata_1537 | 7 | 4 | 7 | 4 | 7 | 6 | 5 | 7 |
| Wollemia_nobilis_13684 | 7 | 4 | 7 | 4 | 7 | 6 | 5 | 7 |
| Dioon_spinulosum_1610 | 7 | 4 | 7 | 4 | 7 | 6 | 5 | 7 |
| Sciadopitys_verticillata_1530 | 7 | 4 | 7 | 4 | 7 | 6 | 5 | 7 |
| **Methionine** | | | | | | | | |
| Retrophyllum_piresii_6159 | 6 | 4 | 9 | 5 | 7 | 5 | 5 | 7 |
| Welwitschia_mirabilis_9156 | 7 | 4 | 9 | 5 | 7 | 5 | 5 | 7 |
| Cycas_debaoensis_37229 | 6 | 4 | 9 | 5 | 7 | 5 | 5 | 7 |
| Ephedra_equisetina_8795 | 6 | 4 | 9 | 5 | 7 | 5 | 5 | 7 |
| Ginkgo_biloba_41168 | 6 | 4 | 9 | 5 | 7 | 5 | 5 | 7 |
| Cunninghamia_lanceolata_17473 | 6 | 4 | 9 | 5 | 7 | 5 | 5 | 7 |
| Dioon_spinulosum_38618 | 6 | 4 | 9 | 5 | 7 | 5 | 5 | 7 |
| Welwitschia_mirabilis_40317 | 7 | 4 | 8 | 5 | 7 | 5 | 5 | 7 |
| Retrophyllum_piresii_21077 | 7 | 4 | 8 | 5 | 7 | 5 | 5 | 7 |
| Cycas_debaoensis_55239 | 7 | 4 | 8 | 5 | 7 | 5 | 5 | 7 |
| Gnetum_gnemon_48648 | 7 | 4 | 8 | 5 | 7 | 5 | 5 | 7 |
| Ephedra_equisetina_36161 | 7 | 4 | 8 | 5 | 7 | 5 | 5 | 7 |
| Cedrus_deodara_28008 | 6 | 4 | 9 | 5 | 7 | 5 | 5 | 7 |
| Ginkgo_biloba_57058 | 7 | 4 | 8 | 5 | 7 | 5 | 5 | 7 |
| Wollemia_nobilis_107496 | 7 | 4 | 8 | 8 | 5 | 7 | 5 | 7 |
| Dioon_spinulosum_56165 | 7 | 4 | 8 | 5 | 7 | 5 | 5 | 7 |
| Sciadopitys_verticillata_70619 | 6 | 4 | 9 | 5 | 7 | 5 | 5 | 7 |
| Taxus_mairei_105676 | 6 | 4 | 9 | 5 | 7 | 5 | 5 | 7 |
| Gnetum_gnemon_76264 | 6 | 4 | 9 | 5 | 7 | 5 | 5 | 7 |
| Cedrus_deodara_69483 | 7 | 4 | 8 | 5 | 7 | 5 | 5 | 7 |
| Cunninghamia_lanceolata_127080 | 7 | 4 | 8 | 5 | 7 | 5 | 5 | 7 |
| Wollemia_nobilis_124427 | 6 | 4 | 9 | 5 | 7 | 5 | 5 | 7 |
| Sciadopitys_verticillata_85812 | 7 | 4 | 8 | 5 | 7 | 5 | 5 | 7 |
| Taxus_mairei_119804 | 6 | 4 | 8 | 5 | 7 | 5 | 5 | 7 |
| **Phenylalanine** | | | | | | | | |
| Wollemia_nobilis_113114 | 7 | 4 | 8 | 5 | 7 | 5 | 5 | 7 |
| Taxus_mairei_115973 | 7 | 4 | 8 | 5 | 7 | 5 | 5 | 7 |
| Sciadopitys_verticillata_81419 | 7 | 4 | 8 | 5 | 7 | 5 | 5 | 7 |
| Ephedra_equisetina_35551 | 7 | 4 | 8 | 5 | 7 | 5 | 5 | 7 |
| Gnetum_gnemon_77512 | 7 | 4 | 8 | 5 | 7 | 5 | 5 | 7 |
| Retrophyllum_piresii_17256 | 7 | 4 | 8 | 5 | 7 | 5 | 5 | 7 |
| Cunninghamia_lanceolata_118115 | 7 | 4 | 8 | 5 | 7 | 5 | 5 | 7 |
| Dioon_spinulosum_49706 | 7 | 4 | 8 | 5 | 7 | 5 | 5 | 7 |
| Cycas_debaoensis_48422 | 7 | 4 | 8 | 5 | 7 | 5 | 5 | 7 |
| Welwitschia_mirabilis_39040 | 7 | 4 | 8 | 5 | 7 | 5 | 5 | 7 |
| Cedrus_deodara_16836 | 7 | 4 | 8 | 5 | 7 | 5 | 5 | 7 |
| Ginkgo_biloba_52283 | 7 | 4 | 8 | 5 | 7 | 5 | 5 | 7 |
| **Proline** | | | | | | | | |
| Welwitschia_mirabilis_90317 | 7 | 4 | 9 | 5 | 7 | 5 | 5 | 7 |
| Taxus_mairei_50424 | 7 | 4 | 9 | 5 | 7 | 5 | 5 | 7 |
| Retrophyllum_piresii_72305 | 7 | 4 | 9 | 5 | 7 | 5 | 5 | 7 |
| Cycas_debaoensis_118574 | 7 | 4 | 9 | 5 | 7 | 5 | 5 | 7 |
| Gnetum_gnemon_27701 | 7 | 4 | 9 | 5 | 7 | 5 | 5 | 7 |
| Cedrus_deodara_54990 | 7 | 4 | 9 | 5 | 7 | 5 | 5 | 7 |
| Ginkgo_biloba_134743 | 7 | 4 | 9 | 5 | 7 | 5 | 5 | 7 |
| Cunninghamia_lanceolata_103021 | 7 | 4 | 9 | 5 | 7 | 5 | 5 | 7 |
| Wollemia_nobilis_52968 | 7 | 4 | 9 | 5 | 7 | 5 | 5 | 7 |
| Dioon_spinulosum_118622 | 7 | 4 | 9 | 5 | 7 | 5 | 5 | 7 |
| Welwitschia_mirabilis_50230 | 7 | 4 | 10 | 5 | 7 | 5 | 5 | 7 |
| Taxus_mairei_14185 | 7 | 4 | 9 | 5 | 7 | 5 | 5 | 7 |
| Retrophyllum_piresii_35886 | 7 | 4 | 9 | 5 | 7 | 5 | 5 | 7 |
| Cycas_debaoensis_69803 | 7 | 4 | 9 | 5 | 7 | 5 | 5 | 7 |
| Gnetum_gnemon_66942 | 7 | 4 | 10 | 5 | 7 | 5 | 5 | 7 |
| Ephedra_equisetina_44732 | 7 | 4 | 10 | 5 | 7 | 5 | 5 | 7 |
| Cedrus_deodara_115611 | 7 | 4 | 9 | 5 | 7 | 5 | 5 | 7 |
| Ginkgo_biloba_71928 | 7 | 4 | 9 | 5 | 7 | 5 | 5 | 7 |
| Cunninghamia_lanceolata_91064 | 7 | 4 | 9 | 5 | 7 | 5 | 5 | 7 |
| Wollemia_nobilis_91130 | 7 | 4 | 9 | 5 | 7 | 5 | 5 | 7 |
| Dioon_spinulosum_69973 | 7 | 4 | 9 | 5 | 7 | 5 | 5 | 7 |
| Sciadopitys_verticillata_56418 | 7 | 4 | 9 | 5 | 7 | 5 | 5 | 7 |
| **Serine** | | | | | | | | |
| Cedrus_deodara_78705 | 7 | 4 | 26 | 4 | 7 | 5 | 5 | 7 |
| Welwitschia_mirabilis_34874 | 7 | 4 | 26 | 4 | 7 | 5 | 5 | 7 |
| Retrophyllum_piresii_113218 | 7 | 4 | 26 | 4 | 7 | 5 | 5 | 7 |
| Ginkgo_biloba_10910 | 7 | 4 | 26 | 4 | 7 | 5 | 5 | 7 |
| Wollemia_nobilis_6512 | 7 | 4 | 26 | 4 | 7 | 5 | 5 | 7 |
| Welwitschia_mirabilis_36132 | 7 | 3 | 11 | 5 | 7 | 20 | 5 | 7 |
| Taxus_mairei_80259 | 7 | 3 | 11 | 5 | 7 | 7 | 5 | 7 |
| Retrophyllum_piresii_77636 | 7 | 3 | 11 | 5 | 7 | 7 | 5 | 7 |
| Cycas_debaoensis_9648 | 7 | 3 | 11 | 5 | 7 | 19 | 5 | 7 |
| Gnetum_gnemon_80232 | 7 | 3 | 11 | 4 | 9 | 20 | 5 | 7 |
| Ephedra_equisetina_33456 | 7 | 3 | 11 | 5 | 7 | 20 | 5 | 7 |
| Cedrus_deodara_1021 | 7 | 3 | 11 | 5 | 7 | 7 | 5 | 7 |
| Ginkgo_biloba_9881 | 7 | 3 | 11 | 5 | 7 | 19 | 5 | 7 |
| Cunninghamia_lanceolata_44535 | 7 | 3 | 11 | 5 | 7 | 7 | 5 | 7 |
| Wollemia_nobilis_8172 | 7 | 3 | 11 | 5 | 7 | 7 | 5 | 7 |
| Dioon_spinulosum_9377 | 7 | 3 | 11 | 5 | 7 | 7 | 5 | 7 |
| Sciadopitys_verticillata_92815 | 7 | 3 | 11 | 5 | 7 | 7 | 5 | 7 |
| Cycas_debaoensis_45685 | 7 | 3 | 11 | 5 | 7 | 18 | 5 | 7 |
| Gnetum_gnemon_99522 | 7 | 3 | 11 | 5 | 7 | 18 | 5 | 7 |
| Ephedra_equisetina_16107 | 7 | 3 | 11 | 5 | 7 | 18 | 5 | 7 |
| Cunninghamia_lanceolata_9091 | 7 | 3 | 11 | 5 | 7 | 18 | 5 | 7 |
| Wollemia_nobilis_115942 | 7 | 3 | 11 | 5 | 7 | 18 | 5 | 7 |
| Dioon_spinulosum_47009 | 7 | 3 | 11 | 5 | 7 | 18 | 5 | 7 |
| Sciadopitys_verticillata_78768 | 7 | 3 | 11 | 5 | 7 | 18 | 5 | 7 |
| Welwitschia_mirabilis_8394 | 7 | 4 | 9 | 5 | 7 | 17 | 5 | 7 |
| Taxus_mairei_104821 | 7 | 4 | 9 | 5 | 7 | 19 | 5 | 7 |
| Retrophyllum_piresii_5103 | 7 | 4 | 9 | 5 | 7 | 18 | 5 | 8 |
| Cycas_debaoensis_36209 | 7 | 4 | 9 | 5 | 7 | 20 | 5 | 7 |
| Gnetum_gnemon_107957 | 7 | 4 | 9 | 5 | 7 | 16 | 5 | 7 |
| Ephedra_equisetina_8158 | 7 | 4 | 9 | 5 | 7 | 18 | 5 | 7 |
| Cedrus_deodara_29035 | 7 | 4 | 9 | 5 | 7 | 18 | 5 | 7 |
| Ginkgo_biloba_40045 | 7 | 4 | 9 | 5 | 7 | 21 | 5 | 7 |
| Cunninghamia_lanceolata_18394 | 7 | 4 | 9 | 5 | 7 | 18 | 5 | 7 |
| Wollemia_nobilis_125484 | 7 | 4 | 9 | 5 | 7 | 15 | 5 | 7 |
| Dioon_spinulosum_37484 | 7 | 4 | 9 | 5 | 7 | 20 | 5 | 7 |
| Sciadopitys_verticillata_69531 | 7 | 4 | 9 | 5 | 7 | 18 | 5 | 7 |
| **Threonine** | | | | | | | | |
| Gnetum_gnemon_80858 | 7 | 3 | 15 | 4 | 7 | 5 | 7 | 1 |
| Cunninghamia_lanceolata_42938 | 7 | 4 | 8 | 1 | 7 | 8 | 5 | 7 |
| Cycas_debaoensis_10684 | 7 | 4 | 8 | 5 | 7 | 5 | 5 | 7 |
| Welwitschia_mirabilis_5065 | 7 | 4 | 7 | 5 | 7 | 5 | 5 | 7 |
| Taxus_mairei_100844 | 7 | 4 | 7 | 5 | 7 | 5 | 5 | 7 |
| Retrophyllum_piresii_1314 | 7 | 4 | 7 | 5 | 7 | 5 | 5 | 7 |
| Ephedra_equisetina_5370 | 7 | 4 | 7 | 5 | 7 | 5 | 5 | 7 |
| Cedrus_deodara_100375 | 7 | 4 | 7 | 5 | 7 | 5 | 5 | 7 |
| Ginkgo_biloba_35960 | 7 | 4 | 7 | 5 | 7 | 5 | 5 | 7 |
| Wollemia_nobilis_129602 | 7 | 4 | 7 | 5 | 7 | 5 | 5 | 7 |
| Dioon_spinulosum_33411 | 7 | 3 | 9 | 5 | 7 | 5 | 5 | 7 |
| Sciadopitys_verticillata_65711 | 7 | 4 | 7 | 5 | 7 | 5 | 5 | 7 |
| Sciadopitys_verticillata_94443 | 7 | 3 | 9 | 5 | 9 | 3 | 5 | 7 |
| Welwitschia_mirabilis_38002 | 7 | 4 | 8 | 5 | 7 | 5 | 5 | 7 |
| Taxus_mairei_114516 | 7 | 4 | 8 | 5 | 7 | 5 | 5 | 7 |
| Retrophyllum_piresii_15886 | 7 | 4 | 8 | 5 | 7 | 5 | 5 | 7 |
| Gnetum_gnemon_78449 | 7 | 4 | 8 | 5 | 7 | 5 | 5 | 7 |
| Cedrus_deodara_18275 | 7 | 4 | 8 | 5 | 7 | 5 | 5 | 7 |
| Ginkgo_biloba_50879 | 7 | 4 | 8 | 5 | 7 | 5 | 5 | 7 |
| Cunninghamia_lanceolata_7850 | 7 | 4 | 8 | 5 | 7 | 5 | 5 | 7 |
| Wollemia_nobilis_114545 | 7 | 4 | 8 | 5 | 7 | 5 | 5 | 7 |
| Dioon_spinulosum_48239 | 7 | 4 | 8 | 5 | 7 | 5 | 5 | 7 |
| **Tryptophan** | | | | | | | | |
| Welwitschia_mirabilis_50083 | 7 | 4 | 9 | 5 | 7 | 5 | 5 | 7 |
| Taxus_mairei_14035 | 7 | 4 | 9 | 5 | 7 | 5 | 5 | 7 |
| Retrophyllum_piresii_35651 | 7 | 4 | 9 | 5 | 7 | 5 | 5 | 7 |
| Cycas_debaoensis_69559 | 7 | 4 | 9 | 5 | 7 | 5 | 5 | 7 |
| Gnetum_gnemon_67124 | 7 | 4 | 9 | 5 | 7 | 5 | 5 | 7 |
| Cedrus_deodara_115364 | 7 | 4 | 9 | 5 | 7 | 5 | 5 | 7 |
| Ginkgo_biloba_71691 | 7 | 4 | 9 | 5 | 7 | 5 | 5 | 7 |
| Cunninghamia_lanceolata_90841 | 7 | 4 | 9 | 5 | 7 | 5 | 5 | 7 |
| Wollemia_nobilis_90887 | 7 | 4 | 9 | 5 | 7 | 5 | 5 | 7 |
| Dioon_spinulosum_69732 | 7 | 4 | 9 | 5 | 7 | 5 | 5 | 7 |
| Sciadopitys_verticillata_56651 | 7 | 4 | 9 | 5 | 7 | 5 | 5 | 7 |
| Ephedra_equisetina_40115 | 7 | 4 | 9 | 5 | 7 | 5 | 5 | 7 |
| Ephedra_equisetina_44586 | 7 | 4 | 9 | 5 | 7 | 6 | 4 | 7 |
| **Tyrosine** | | | | | | | | |
| Ginkgo_biloba_29738 | 6 | 3 | 9 | 5 | 7 | 11 | 5 | 7 |
| Cedrus_deodara_101419 | 7 | 4 | 9 | 4 | 29 | 6 | 5 | 7 |
| Wollemia_nobilis_107737 | 7 | 4 | 9 | 4 | 29 | 6 | 5 | 7 |
| Ginkgo_biloba_56265 | 7 | 4 | 9 | 4 | 29 | 6 | 5 | 7 |
| Welwitschia_mirabilis_37523 | 7 | 4 | 9 | 5 | 7 | 16 | 5 | 7 |
| Taxus_mairei_99746 | 7 | 4 | 9 | 5 | 7 | 15 | 5 | 7 |
| Retrophyllum_piresii_152 | 7 | 4 | 9 | 5 | 7 | 16 | 5 | 7 |
| Cycas_debaoensis_31939 | 7 | 4 | 9 | 5 | 7 | 15 | 5 | 7 |
| Gnetum_gnemon_78740 | 7 | 4 | 9 | 5 | 7 | 16 | 5 | 7 |
| Ephedra_equisetina_34413 | 7 | 4 | 9 | 5 | 7 | 15 | 5 | 7 |
| Cunninghamia_lanceolata_22925 | 7 | 4 | 9 | 5 | 7 | 4 | 5 | 7 |
| Dioon_spinulosum_32134 | 7 | 4 | 9 | 5 | 7 | 15 | 5 | 7 |
| Sciadopitys_verticillata_64404 | 7 | 4 | 9 | 5 | 7 | 4 | 5 | 7 |
| **Valine** | | | | | | | | |
| Welwitschia_mirabilis_79656 | 7 | 4 | 7 | 5 | 7 | 5 | 5 | 7 |
| Cycas_debaoensisv_104248 | 7 | 4 | 7 | 5 | 7 | 5 | 5 | 7 |
| Gnetum_gnemon_11709 | 7 | 4 | 7 | 5 | 7 | 5 | 5 | 7 |
| Ephedra_equisetina_70330 | 7 | 4 | 7 | 5 | 7 | 5 | 5 | 7 |
| Cedrus_deodara_57402 | 7 | 4 | 7 | 5 | 7 | 5 | 5 | 7 |
| Ginkgo_biloba_107181 | 7 | 4 | 7 | 5 | 7 | 5 | 5 | 7 |
| Cunninghamia_lanceolata_55579 | 7 | 4 | 7 | 5 | 7 | 5 | 5 | 7 |
| Wollemia_nobilis_36714 | 7 | 4 | 7 | 5 | 7 | 5 | 5 | 7 |
| Dioon_spinulosum_146145 | 7 | 4 | 7 | 5 | 7 | 5 | 5 | 7 |
| Sciadopitys_verticillata_18221 | 7 | 4 | 7 | 5 | 7 | 5 | 5 | 7 |
| Retrophyllum_piresii_103285 | 7 | 4 | 7 | 5 | 7 | 5 | 5 | 7 |
| Welwitschia_mirabilis_82684 | 7 | 4 | 8 | 4 | 7 | 6 | 5 | 7 |
| Retrophyllum_piresii_20310 | 7 | 4 | 9 | 4 | 7 | 6 | 5 | 7 |
| Cycas_debaoensis_54436 | 7 | 4 | 9 | 4 | 7 | 6 | 5 | 7 |
| Gnetum_gnemon_76485 | 7 | 4 | 8 | 4 | 7 | 6 | 5 | 7 |
| Cunninghamia_lanceolata_127319 | 7 | 4 | 9 | 4 | 7 | 6 | 5 | 7 |
| Dioon_spinulosum_55383 | 7 | 4 | 9 | 4 | 7 | 6 | 5 | 7 |
| Sciadopitys_verticillata_85008 | 7 | 4 | 9 | 4 | 7 | 6 | 5 | 7 |
