# Supplementary material for: Evolutionary analysis of chloroplast tRNA of Gymnosperm revealed the novel structural variation and evolutionary aspect
Source: PeerJ. 2020 Nov 25;8:e10312. doi: 10.7717/peerj.10312 (PMC7698693; doi:10.7717/peerj.10312)
Supplement: Supplemental Information 6 — 220 loss events and 153 duplication events are detected in gymnosperm chloroplast tRNAs, and loss events have occurred slightly more frequently than duplication events. [file peerj-08-10312-s006.docx]

| Table S2 Loss events of chloroplast genomic tRNAs. | | | | |
| --- | --- | --- | --- | --- |
| **tRNA Gene ID** | **Species** | **tRNA** | **Anti-codon** | **Lost in species** |
| 34443 | *G.gnemon* | Ala | UGC | *D.spinulosum* |
| 142660 | *C.debaoensis* | Ala | UGC |  |
| 96165 | *E.equisetina* | Ala | UGC | *G.biloba,W.mirabilis* |
| 82685 | *W.mirabilis* | Ala | UGC | *C.debaoensis* |
| 107167 | *D.spinulosum* | Ala | UGC |  |
| 145059 | *G.biloba* | Ala | UGC | *G.gnemon, E.equisetina* |
| 41240 | *W.nobilis* | Ala | UGC | *R.piresii* |
| 50692 | *C.lanceolata* | Ala | UGC | *T.mairei,S.verticillata* |
| 12642 | *S.verticillata* | Ala | UGC | *T.mairei,W.nobilis,R.piresii,G.gnemon,E.equisetina,C.deodara,G.biloba,C.debaoensis,W.mirabilis* |
| 90492 | *S.verticillata* | Arg | CCG | *T.mairei,C.lanceolata,G.biloba* |
| 46913 | *C.lanceolata* | Arg | ACG | *T.mairei,S.verticillata* |
| 19249 | *G.gnemon* | Arg | ACG | *E.equisetina,C.deodara,G.biloba,W.mirabilis,C.debaoensis* |
| 8524 | *S.verticillata* | Arg | ACG | *T.mairei,C.lanceolata* |
| 118622 | *D.spinulosum* | Arg | ACG | *C.debaoensis* |
| 111990 | *C.debaoensis* | Arg | ACG | *D.spinulosum,S.verticillata,C.lanceolata,T.mairei,C.deodara,G.biloba,G.gnemon* |
| 45150 | *T.mairei* | Arg | ACG | *C.lanceolata,S.verticillata,G.gnemon,E.equisetina,C.deodara,G.biloba,W.mirabilis,C.debaoensis* |
| 46496 | *W.nobilis* | Arg | ACG | *R.piresii* |
| 112715 | *R.piresii* | Arg | ACG | *W.nobilis,G.gnemon,E.equisetina,S.verticillata,C.lanceolata,T.mairei,C.deodara,G.biloba,W.mirabilis,C.debaoensis* |
| 42688 | *C.lanceolata* | Arg | UCU | *T.mairei,S.verticillata,G.gnemon* |
| 81144 | *T.mairei* | Arg | UCU | *C.lanceolata,S.verticillata,E.equisetina,W.mirabilis* |
| 34644 | *W.mirabilis* | Arg | UCU | *R.piresii* |
| 11752 | *C.debaoensis* | Arg | UCU | *D.spinulosum* |
| 11473 | *D.spinulosum* | Arg | UCU | *C.debaoensis,C.deodara,W.nobilis,R.piresii* |
| 114287 | *R.piresii* | Arg | UCU | *W.nobilis,E.equisetina,G.gnemon,C.deodara,W.nobilis,R.piresii,G.biloba,W.mirabilis,C.debaoensis, D.spinulosum* |
| 90759 | *S.verticillata* | Arg | UCU | *T.mairei,C.lanceolata,E.equisetina,G.gnemon,C.deodara,W.nobilis,R.piresii,G.biloba,W.mirabilis, C.debaoensis,D.spinulosum* |
| 61512 | *G.biloba* | Arg | CCG | *E.equisetina,G.gnemon,S.verticillata,C.lanceolata,T.mairei,C.deodara,C.debaoensis,D.spinulosum, W.mirabilis* |
| 77973 | *E.equisetina* | Asn | GUU | *T.mairei,S.verticillata,C.deodara,W.nobilis,R.piresii,G.gnemon,W.mirabilis,C.debaoensis* |
| 19530 | *G.gnemon* | Asn | GUU |  |
| 46290 | *C.lanceolata* | Asn | GUU |  |
| 100730 | *W.mirabilis* | Asn | GUU | *T.mairei,C.lanceolata* |
| 8201 | *S.verticillata* | Asn | GUU |  |
| 47307 | *W.nobilis* | Asn | GUU | *R.piresii* |
| 138071 | *C.debaoensis* | Asn | GUU | *D.spinulosum* |
| 137554 | *D.spinulosum* | Asn | GUU | *C.debaoensis,W.nobilis,R.piresii,G.biloba* |
| 76577 | *R.piresii* | Asn | GUU | *W.nobilis,C.deodara,G.biloba,C.debaoensis,D.spinulosum* |
| 45672 | *T.mairei* | Asn | GUU | *C.lanceolata,S.verticillata,C.deodara,W.nobilis,R.piresii,G.biloba,W.mirabilis,C.debaoensis, D.spinulosum,E.equisetina,G.gnemon* |
| 46290 | *C.lanceolata* | Asn | GUU | *T.mairei,S.verticillata,E.equisetina,G.gnemon,C.deodara,W.nobilis,R.piresii,G.biloba,W.mirabilis, C.debaoensis,D.spinulosum* |
| 25574 | *R.piresii* | Asp | GUC | *W.nobilis,S.verticillata,C.lanceolata,T.mairei,C.deodara,G.biloba* |
| 133138 | *R.piresii* | Asp | GUC |  |
| 71649 | *W.nobilis* | Asp | GUC | *R.piresii* |
| 23222 | *C.lanceolata* | Asp | GUC | *T.mairei,S.verticillata* |
| 99499 | *T.mairei* | Asp | GUC | *C.lanceolata,S.verticillata,W.nobilis,R.piresii* |
| 64130 | *S.verticillata* | Asp | GUC | *T.mairei,C.lanceolata,C.deodara,W.nobilis,R.piresii,G.biloba* |
| 31761 | *D.spinulosum* | Asp | GUC | *C.debaoensis* |
| 31566 | *C.debaoensis* | Asp | GUC | *D.spinulosum,S.verticillata,C.lanceolata,T.mairei,C.deodara,W.nobilis,R.piresii,G.biloba* |
| 37752 | *W.mirabilis* | Asp | GUC | *S.verticillata,C.lanceolata,T.mairei,C.deodara,W.nobilis,R.piresii,G.biloba,C.debaoensis,D.spinulosum* |
| 78589 | *G.gnemon* | Asp | GUC |  |
| 34603 | *E.equisetina* | Asp | GUC |  |
| 137519 | *S.verticillata* | Cys | GCA | *T.mairei,C.lanceolata* |
| 134757 | *W.nobilis* | Cys | GCA | *R.piresii,G.gnemon,S.verticillata,C.lanceolata,T.mairei,W.mirabilisv* |
| 97135 | *T.mairei* | Cys | GCA | *C.lanceolata,S.verticillata,G.biloba,W.nobilis,R.piresii,W.mirabilisv* |
| 26571 | *C.lanceolata* | Cys | GCA | *T.mairei,S.verticillata,G.gnemon,W.nobilis,R.piresii,W.mirabilis* |
| 29928 | *G.biloba* | Cys | ACA | *G.gnemon,S.verticillata,C.lanceolata,T.mairei,C.lanceolata,W.nobilis,R.piresii,C.debaoensis, D.spinulosum,W.mirabilis* |
| 108018 | *R.piresii* | Cys | GCA | *W.nobilis,S.verticillata,C.lanceolata,T.mairei* |
| 8211 | *G.biloba* | Gln | UUG | *E.equisetina,G.gnemon,S.verticillata,C.lanceolata,T.mairei,C.deodara,W.nobilis,R.piresii,W.mirabilis,C.debaoensis,D.spinulosum* |
| 37177 | *W.mirabilis* | Gln | UUG | *E.equisetina,G.gnemon,S.verticillata,C.lanceolata,T.mairei,C.deodara,W.nobilis,R.piresii,G.biloba, C.debaoensis,D.spinulosum* |
| 76449 | *C.deodara* | Gln | UUG | *E.equisetina,G.gnemon,S.verticillata,C.lanceolata,T.mairei,W.nobilis,R.piresii,G.biloba,W.mirabilis, C.debaoensis,D.spinulosum* |
| 7818 | *D.spinulosum* | Gln | UUG | *E.equisetina,G.gnemon,S.verticillata,C.lanceolata,T.mairei,C.deodara,W.nobilis,R.piresii,G.biloba, W.mirabilis,C.debaoensis,D.spinulosum* |
| 8078 | *C.debaoensis* | Gln | UUG |  |
| 9806 | *W.nobilis* | Gln | UUG | *R.piresii,E.equisetina,G.gnemon,S.verticillata,C.lanceolata,T.mairei,C.deodara,G.biloba,W.mirabilis,C.debaoensis,D.spinulosum* |
| 46041 | *C.lanceolata* | Gln | UUG | *T.mairei,S.verticillata,E.equisetina,G.gnemon,S.verticillata,C.lanceolata,T.mairei,W.nobilis,R.piresii,G.biloba,W.mirabilis,C.debaoensis,D.spinulosum* |
| 94297 | *S.verticillata* | Gln | UUG | *C.lanceolata,E.equisetina,G.gnemon,S.verticillata,C.lanceolata,T.mairei,W.nobilis,R.piresii,G.biloba,W.mirabilis,C.debaoensis,D.spinulosum* |
| 94370 | *S.verticillata* | Gln | UUG |  |
| 78840 | *T.mairei* | Gln | UUG |  |
| 79005 | *R.piresii* | Gln | UUG | *W.nobilis,E.equisetina,G.gnemon,S.verticillata,C.lanceolata,T.mairei,C.deodara,G.biloba,W.mirabilis,C.debaoensis,D.spinulosum* |
| 34120 | *E.equisetina* | Gln | UUG | *G.gnemon,S.verticillata,C.lanceolata,T.mairei,C.deodara,W.nobilis,R.piresii,G.biloba,W.mirabilis, C.debaoensis,D.spinulosum* |
| 7625 | *C.lanceolata* | Gln | UUG | *T.mairei,S.verticillata,E.equisetina,G.gnemon,W.mirabilis,C.deodara,W.nobilis,R.piresii,G.biloba, C.debaoensis,D.spinulosum* |
| 34120 | *G.gnemon* | Gln | UUG | *E.equisetina,S.verticillata,C.lanceolata,T.mairei,C.deodara,W.nobilis,R.piresii,G.biloba,W.mirabilis, C.debaoensis,D.spinulosum* |
| 106909 | *R.piresii* | Glu | UUC | *W.nobilis,W.mirabilis* |
| 130551 | *W.nobilis* | Glu | UUC | *R.piresii,G.gnemon* |
| 13581 | *S.verticillata* | Glu | UUC | *T.mairei,C.lanceolata* |
| 143585 | *C.debaoensis* | Glu | UUC | *D.spinulosum,E.equisetina,C.deodara,G.biloba,W.nobilis,R.piresii,G.biloba,W.mirabilis,C.debaoensis,D.spinulosum* |
| 143118 | *D.spinulosum* | Glu | UUC | *C.debaoensis* |
| 51599 | *C.lanceolata* | Glu | UUC | *T.mairei,S.verticillata* |
| 35405 | *C.deodara* | Glu | UUC | *W.nobilis,R.piresii,G.gnemon,W.mirabilis* |
| 32282 | *D.spinulosum* | Glu | UUC | *C.debaoensis* |
| 32087 | *C.debaoensis* | Glu | UUC | *D.spinulosum,E.equisetina,G.biloba,W.mirabilis* |
| 22791 | *C.lanceolata* | Glu | UUC | *S.verticillata* |
| 99898 | *T.mairei* | Glu | UUC |  |
| 64546 | *S.verticillata* | Glu | UUC | *T.mairei,C.lanceolata,E.equisetina,G.gnemon,C.deodara,G.biloba,W.nobilis,R.piresii,W.mirabilis, C.debaoensis,D.spinulosum* |
| 17651 | *C.lanceolata* | Gly | GCC | *T.mairei,S.verticillata* |
| 37035 | *C.debaoensis* | Gly | GCC | *D.spinulosum* |
| 70419 | *S.verticillata* | Gly | GCC | *T.mairei,C.lanceolata* |
| 38421 | *D.spinulosum* | Gly | GCC | *C.debaoensis,G.gnemon,E.equisetina,C.deodara,W.mirabilis* |
| 5971 | *R.piresii* | Gly | GCC | *W.nobilis* |
| 124623 | *W.nobilis* | Gly | GCC | *R.piresii,G.biloba* |
| 90178 | *W.nobilis* | Gly | UCC | *R.piresii,G.gnemon,E.equisetina,S.verticillata,C.lanceolata,T.mairei,C.deodara,G.biloba,W.mirabilis,C.debaoensis,D.spinulosum* |
| 105489 | *T.mairei* | Gly | GCC | *C.lanceolata,S.verticillata,E.equisetina,G.gnemon,C.deodara,W.nobilis,R.piresii,G.biloba,W.mirabilis,C.debaoensis,D.spinulosum* |
| 161222 | *D.spinulosum* | His | GUG | *C.debaoensis* |
| 18545 | *W.nobilis* | His | GUG | *R.piresii* |
| 161706 | *C.debaoensis* | His | GUG | *D.spinulosum,G.biloba,W.nobilis,R.piresii,E.equisetina,G.gnemon,S.verticillata,C.lanceolata,T.mairei,W.mirabilis,C.debaoensis* |
| 1622 | *G.gnemon* | His | GUG | *S.verticillata,C.lanceolata,T.mairei,C.deodara,G.biloba,W.nobilis,R.piresii,W.mirabilis,C.debaoensis* |
| 118920 | *W.mirabilis* | His | GUG | *G.gnemon,E.equisetina,S.verticillata,C.lanceolata,T.mairei,C.deodara,G.biloba,W.nobilis,R.piresii, C.debaoensis* |
| 69151 | *C.deodara* | His | GUG | *E.equisetina,G.gnemon,S.verticillata,C.lanceolata,T.mairei,G.biloba,W.nobilis,R.piresii,W.mirabilis, C.debaoensis* |
| 120805 | *S.verticillata* | His | GUG | *T.mairei,C.lanceolata,C.deodara,E.equisetina,G.gnemon,C.deodara,G.biloba,W.nobilis,R.piresii, W.mirabilis,C.debaoensis* |
| 24 | *G.biloba* | His | GUG | *E.equisetina,G.gnemon,S.verticillata,C.lanceolata,T.mairei,C.deodara,G.biloba,W.nobilis,R.piresii, C.debaoensis,W.mirabilis* |
| 86732 | *R.piresii* | His | GUG | *W.nobilis,E.equisetina,G.gnemon,S.verticillata,C.lanceolata,T.mairei,C.deodara,G.biloba,W.mirabilis,C.debaoensis,D.spinulosum* |
| 134766 | *C.lanceolata* | His | GUG | *S.verticillata,E.equisetina,G.gnemon,W.mirabilis* |
| 71194 | *T.mairei* | His | GUG |  |
| 119109 | *T.mairei* | Ile | UAU | *C.lanceolata,C.deodara,S.verticillata,C.deodara,S.verticillata,C.lanceolata,T.mairei,G.biloba, C.debaoensis,D.spinulosum,E.equisetina,G.gnemon,W.mirabilis* |
| 80565 | *S.verticillata* | Leu | UAA | *T.mairei,C.lanceolata* |
| 117276 | *C.lanceolata* | Leu | UAA | *T.mairei,S.verticillata,G.gnemon,E.equisetina,W.nobilis,R.piresii,W.mirabilis* |
| 115126 | *T.mairei* | Leu | UAA | *C.lanceolata,S.verticillata,G.gnemon,E.equisetina,W.nobilis,R.piresii,W.mirabilis* |
| 97776 | *R.piresii* | Leu | CAA | *W.nobilis,G.gnemon,S.verticillata,C.lanceolata,T.mairei,G.biloba,W.mirabilis,C.debaoensis, D.spinulosum* |
| 27863 | *G.gnemon* | Leu | UAG | *W.mirabilis,E.equisetina,S.verticillata,C.lanceolata,T.mairei,C.deodara,W.nobilis,R.piresii,G.biloba, C.debaoensis,D.spinulosum* |
| 30036 | *W.nobilis* | Leu | CAA | *R.piresii* |
| 8867 | *G.gnemon* | Leu | CAA | *E.equisetina* |
| 61986 | *C.debaoensis* | Leu | UAG | *D.spinulosum,S.verticillata,C.lanceolata,T.mairei,W.nobilis,R.piresii,G.biloba,W.mirabilis* |
| 90133 | *W.mirabilis* | Leu | UAG | *T.mairei,C.lanceolata* |
| 117202 | *S.verticillata* | Leu | UAG |  |
| 50656 | *T.mairei* | Leu | UAG | *C.lanceolata,S.verticillata,C.deodara,W.mirabilis,W.nobilis,R.piresii,G.biloba,C.debaoensis, D.spinulosum* |
| 118837 | *D.spinulosum* | Leu | UAG | *C.debaoensis* |
| 53167 | *W.nobilis* | Leu | UAG | *R.piresii* |
| 103246 | *C.lanceolata* | Leu | UAG | *T.mairei,S.verticillata* |
| 72106 | *R.piresii* | Leu | UAG | *W.nobilis,S.verticillata,C.lanceolata,T.mairei,C.debaoensis,D.spinulosum* |
| 134523 | *G.biloba* | Leu | UAG | *C.deodara,W.mirabilis,E.equisetina,G.gnemon* |
| 73018 | *T.mairei* | Lys | UUU | *C.lanceolata,S.verticillata,E.equisetina,C.lanceolata,* |
| 1537 | *C.lanceolata* | Lys | UUU | *T.mairei,S.verticillata,E.equisetina,G.gnemon,C.deodara,W.nobilis,R.piresii,G.biloba,W.mirabilis, C.debaoensis,D.spinulosum* |
| 112476 | *G.gnemon* | Lys | UUU | *E.equisetina,S.verticillata,C.lanceolata,T.mairei,C.deodara,W.nobilis,R.piresii,G.biloba,C.debaoensis,D.spinulosum* |
| 1209 | *E.equisetina* | Lys | UUU | *G.gnemon,S.verticillata,C.lanceolata,T.mairei,C.deodara,W.nobilis,R.piresii,G.biloba,W.mirabilis, C.debaoensis,D.spinulosum* |
| 1530 | *S.verticillata* | Lys | UUU | *T.mairei,C.lanceolata* |
| 13684 | *W.nobilis* | Lys | UUU | *R.piresii* |
| 82037 | *R.piresii* | Lys | UUU | *W.nobilis,S.verticillata,C.lanceolata,T.mairei* |
| 1941 | *G.biloba* | Lys | UUU | *D.spinulosum* |
| 1716 | *C.debaoensis* | Lys | UUU |  |
| 1610 | *D.spinulosum* | Lys | UUU | *C.debaoensis,G.biloba,E.equisetina* |
| 38618 | *D.spinulosum* | Met | CAU | *C.debaoensis* |
| 37229 | *C.debaoensis* | Met | CAU | *D.spinulosum,G.biloba* |
| 70619 | *S.verticillata* | Met | CAU | *T.mairei,C.lanceolata* |
| 17473 | *C.lanceolata* | Met | CAU | *S.verticillata* |
| 105676 | *T.mairei* | Met | CAU | *G.gnemon,E.equisetina,C.deodara,G.biloba,W.mirabilis,C.debaoensis* |
| 21077 | *R.piresii* | Met | CAU | *W.nobilis* |
| 88511 | *D.spinulosum* | Met | CAU | *S.verticillata,C.lanceolata,T.mairei,C.deodara，W.nobilis,R.piresii* |
| 88785 | *C.debaoensis* | Met | CAU |  |
| 68903 | *W.mirabilis* | Met | CAU |  |
| 1264 | *G.gnemon* | Met | CAU |  |
| 60791 | *E.equisetina* | Met | CAU |  |
| 90908 | *G.biloba* | Met | CAU |  |
| 21954 | *W.nobilis* | Met | CAU | *R.piresii* |
| 71583 | *T.mairei* | Met | CAU | *C.lanceolata,S.verticillata* |
| 90145 | *R.piresii* | Met | CAU | *W.nobilis* |
| 23714 | *T.mairei* | Met | CAU | *C.lanceolata,S.verticillata,W.nobilis,R.piresii* |
| 34364 | *S.verticillata* | Met | CAU | *T.mairei,C.lanceolata,W.nobilis,R.piresii,G.gnemon,E.equisetina,C.deodara,G.biloba,W.mirabilis, C.debaoensis,D.spinulosum* |
| 40317 | *W.mirabilis* | Met | CAU | *T.mairei,C.lanceolata,C.deodara,W.nobilis,R.piresii,G.biloba* |
| 85812 | *S.verticillata* | Met | CAU |  |
| 127080 | *C.lanceolata* | Met | CAU | *T.mairei,S.verticillata* |
| 119804 | *T.mairei* | Met | CAU | *C.lanceolata,S.verticillata,C.deodara,W.nobilis,R.piresii,G.biloba* |
| 37229 | *C.deodara* | Met | CAU | *G.gnemon,E.equisetina,S.verticillata,C.lanceolata,T.mairei,G.biloba,W.nobilis,R.piresii,W.mirabilis, C.debaoensis,D.spinulosum* |
| 124427 | *W.nobilis* | Met | CAU | *R.piresii,S.verticillata,C.lanceolata,T.mairei,C.deodara,G.biloba,C.debaoensis,D.spinulosum, E.equisetina,G.gnemon,W.mirabilis* |
| 115973 | *T.mairei* | Phe | GAA | *S.verticillata* |
| 49706 | *D.spinulosum* | Phe | GAA | *C.debaoensis* |
| 113114 | *W.nobilis* | Phe | GAA | *R.piresii* |
| 16836 | *C.debaoensis* | Phe | GAA | *D.spinulosum,C.deodara,G.biloba* |
| 17256 | *R.piresii* | Phe | GAA | *W.nobilis,C.deodara,G.biloba,C.debaoensis,D.spinulosum* |
| 81419 | *S.verticillata* | Phe | GAA | *T.mairei,C.lanceolata,C.deodara,W.nobilis,R.piresii,G.biloba,C.debaoensis,D.spinulosum* |
| 91064 | *C.lanceolata* | Pro | UGG | *T.mairei,S.verticillata,* |
| 35886 | *R.piresii* | Pro | UGG | *W.mirabilis* |
| 91130 | *W.nobilis* | Pro | UGG | *R.piresii,C.deodara,S.verticillata,C.lanceolata,T.mairei* |
| 56418 | *S.verticillata* | Pro | UGG | *T.mairei,C.lanceolata* |
| 115611 | *C.deodara* | Pro | UGG | *W.nobilis,R.piresii* |
| 14185 | *T.mairei* | Pro | UGG | *C.lanceolata,S.verticillata,C.deodara,W.nobilis,R.piresii* |
| 50424 | *T.mairei* | Pro | GGG | *C.lanceolata,S.verticillata,C.deodara,W.nobilis,R.piresii,G.biloba* |
| 52968 | *W.nobilis* | Pro | GGG | *R.piresii* |
| 118574 | *C.debaoensis* | Pro | GGG | *D.spinulosum,S.verticillata,C.lanceolata,T.mairei,C.deodara,G.biloba* |
| 103021 | *C.lanceolata* | Pro | GGG | *T.mairei,S.verticillata* |
| 118622 | *D.spinulosum* | Pro | GGG | *C.debaoensis* |
| 72305 | *R.piresii* | Pro | GGG | *W.nobilis,G.gnemon,W.mirabilis,E.equisetina* |
| 9377 | *D.spinulosum* | Ser | GCU | *C.debaoensis,S.verticillata,C.lanceolata,T.mairei,C.deodara,G.biloba,W.nobilis,R.piresii* |
| 45685 | *C.debaoensis* | Ser | GGA | *D.spinulosum* |
| 47009 | *D.spinulosum* | Ser | GGA | *C.debaoensis,S.verticillata,C.lanceolata,T.mairei,C.deodara* |
| 78768 | *S.verticillata* | Ser | GGA | *T.mairei,C.lanceolata,* |
| 115942 | *W.nobilis* | Ser | GGA | *R.piresii* |
| 113218 | *R.piresii* | Ser | CGA | *W.nobilis,W.mirabilis* |
| 10910 | *G.biloba* | Ser | CGA | *G.gnemon,E.equisetina,C.deodara* |
| 22925 | *C.lanceolata* | Ser | UGA | *T.mairei,S.verticillata* |
| 152 | *R.piresii* | Ser | UGA | *W.nobilis,E.equisetina* |
| 64404 | *S.verticillata* | Ser | UGA | *T.mairei,C.lanceolata* |
| 99746 | *T.mairei* | Ser | UGA | *C.lanceolata,S.verticillata,E.equisetina,C.deodara,W.nobilis,R.piresii* |
| 9881 | *G.biloba* | Ser | GCU | *D.spinulosum* |
| 9648 | *C.debaoensis* | Ser | GCU |  |
| 8172 | *W.nobilis* | Ser | GCU | *R.piresii* |
| 77636 | *R.piresii* | Ser | GCU | *W.nobilis,S.verticillata,C.lanceolata,T.mairei* |
| 1021 | *C.deodara* | Ser | GCU | *E.equisetina,G.gnemon,W.mirabilis* |
| 42938 | *C.lanceolata* | Thr | GGU | *T.mairei,S.verticillata* |
| 10684 | *C.debaoensis* | Thr | UGU | *D.spinulosum* |
| 37361 | *T.mairei* | Thr | GGU | *C.lanceolata,S.verticillata,E.equisetina,G.gnemon,C.deodara,W.nobilis,R.piresii,G.biloba,W.mirabilis,C.debaoensis,D.spinulosum* |
| 33411 | *D.spinulosum* | Thr | GGU | *C.debaoensis* |
| 1314 | *R.piresii* | Thr | GGU | *W.nobilis* |
| 129602 | *W.nobilis* | Thr | GGU | *R.piresii,S.verticillata,C.lanceolata,T.mairei,C.deodara,G.biloba,C.debaoensis,D.spinulosum* |
| 100844 | *T.mairei* | Thr | GGU | *C.lanceolata,S.verticillata,C.deodara,G.biloba,W.nobilis,R.piresii,C.debaoensis,D.spinulosum, E.equisetina,G.gnemon,W.mirabilis* |
| 7850 | *C.lanceolata* | Thr | UGU | *T.mairei,S.verticillata* |
| 50879 | *G.biloba* | Thr | UGU | *G.gnemon,W.mirabilis* |
| 78449 | *G.gnemon* | Thr | UGU | *C.lanceolata,S.verticillata* |
| 114516 | *T.mairei* | Thr | UGU |  |
| 38002 | *W.mirabilis* | Thr | UGU | *C.deodara,W.nobilis,R.piresii,G.biloba* |
| 5065 | *W.mirabilis* | Thr | GGU | *S.verticillata,C.lanceolata,T.mairei,C.deodara,W.nobilis,R.piresii,G.biloba,C.debaoensis, D.spinulosum* |
| 35960 | *G.gnemon* | Thr | GGU |  |
| 5370 | *E.equisetina* | Thr | GGU |  |
| 65711 | *S.verticillata* | Thr | GGU | *T.mairei,C.lanceolata* |
| 34824 | *E.equisetina* | Thr | UGU | *G.gnemon,S.verticillata,C.lanceolata,T.mairei,C.deodara,G.biloba,C.debaoensis,D.spinulosum, W.mirabilis* |
| 80858 | *G.gnemon* | Thr | GGU | *E.equisetina,S.verticillata,C.lanceolata,T.mairei,G.biloba,C.debaoensis,W.mirabilis* |
| 14035 | *T.mairei* | Trp | CCA | *C.lanceolata,S.verticillata* |
| 90887 | *W.nobilis* | Trp | CCA | *R.piresii* |
| 35651 | *R.piresii* | Trp | CCA | *W.nobilis,S.verticillata,C.lanceolata,T.mairei,C.deodara,G.biloba,C.debaoensis,D.spinulosum* |
| 56651 | *S.verticillata* | Trp | CCA | *T.mairei,C.lanceolata,C.deodara,W.nobilis,R.piresii,G.biloba,C.debaoensis,D.spinulosum* |
| 78740 | *G.gnemon* | Tyr | GUA | *S.verticillata,C.lanceolata,T.mairei,G.biloba,W.nobilis,R.piresii,C.debaoensis,W.mirabilis* |
| 29738 | *G.biloba* | Tyr | AUA | *G.gnemon,S.verticillata,C.lanceolata,T.mairei,C.lanceolata,W.nobilis,R.piresii,C.debaoensis, D.spinulosum,W.mirabilis* |
| 107737 | *W.nobilis* | Tyr | AUA | *R.piresii* |
| 152 | *R.piresii* | Tyr | GUA | *W.nobilis,S.verticillata,C.lanceolata,T.mairei,C.deodara,G.biloba,C.debaoensis,D.spinulosum, W.mirabilis,E.equisetina,G.gnemon* |
| 146145 | *D.spinulosum* | Val | GAC | *C.debaoensis* |
| 104248 | *C.debaoensis* | Val | GAC | *D.spinulosum,G.biloba* |
| 18221 | *S.verticillata* | Val | GAC | *T.mairei,C.lanceolata,* |
| 55579 | *C.lanceolata* | Val | GAC | *T.mairei,S.verticillata,E.equisetina,G.gnemon,C.deodara,W.nobilis,R.piresii,G.biloba,W.mirabilis, C.debaoensis,D.spinulosum* |
| 39496 | *W.mirabilis* | Val | UAC | *E.equisetina,C.deodara,G.biloba,C.debaoensis,D.spinulosum,S.verticillata,C.lanceolata,T.mairei, W.nobilis,R.piresii* |
| 76485 | *G.gnemon* | Val | UAC |  |
| 85008 | *S.verticillata* | Val | UAC | *T.mairei,C.lanceolata* |
| 55383 | *D.spinulosum* | Val | UAC | *W.nobilis,S.verticillata,C.lanceolata,T.mairei,C.deodara,G.biloba* |
| 54436 | *C.debaoensis* | Val | UAC |  |
| 20310 | *R.piresii* | Val | UAC |  |
| 127319 | *C.lanceolata* | Val | UAC | *T.mairei,S.verticillata,C.lanceolata,T.mairei,C.deodara,G.biloba,C.debaoensis,D.spinulosum* |
