# Supplementary material for: Evolutionary analysis of chloroplast tRNA of Gymnosperm revealed the novel structural variation and evolutionary aspect
Source: PeerJ. 2020 Nov 25;8:e10312. doi: 10.7717/peerj.10312 (PMC7698693; doi:10.7717/peerj.10312)
Supplement: Supplemental Information 4 — The tRNA genes are shown in the left (top to bottom). Boxes in light green, dark green, and white represent one copy of tRNA genes, two copies of tRNA genes, and the absence of tRNA genes. [file peerj-08-10312-s004.pdf]

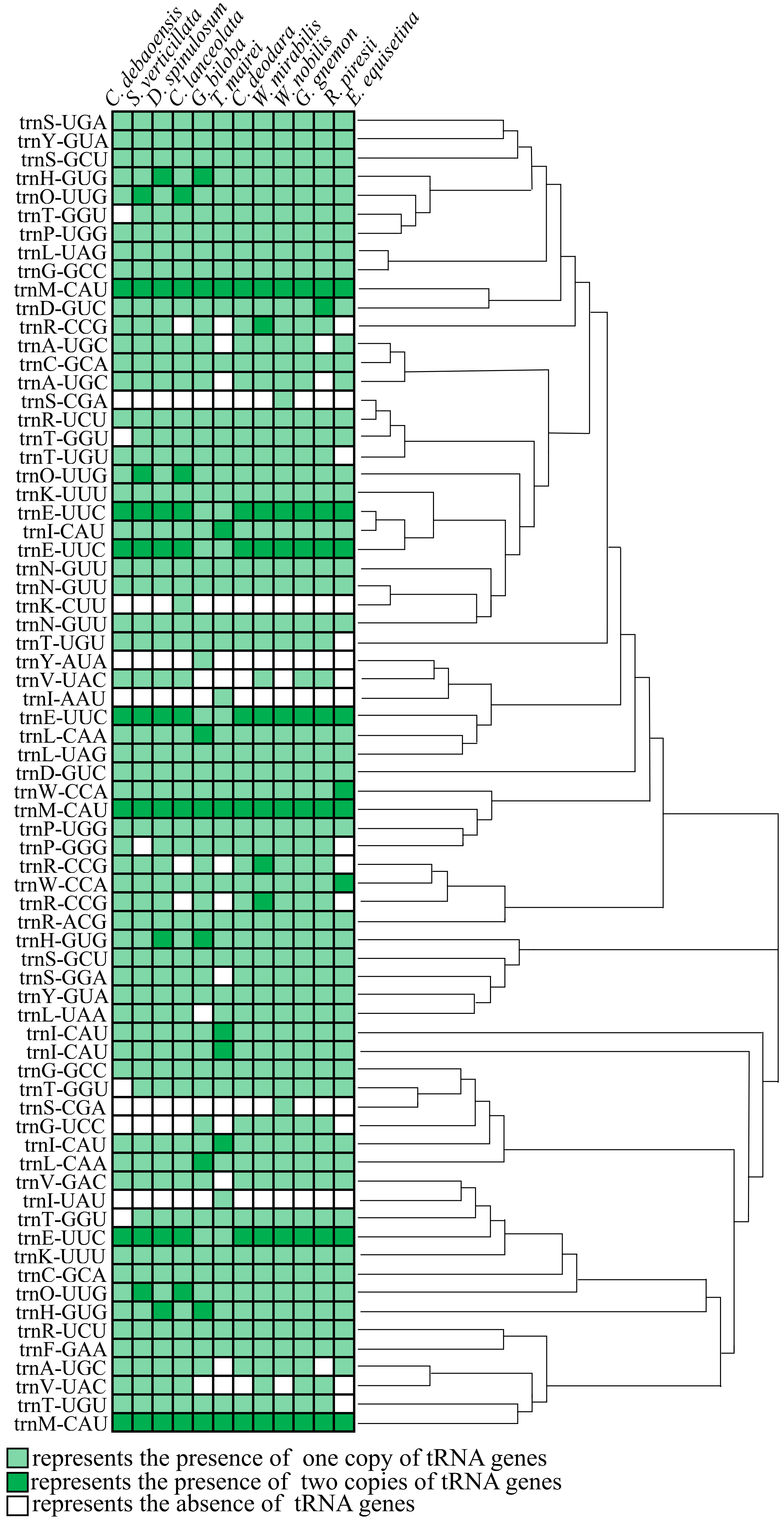
C. debaoensis
S. verticillata
D. spinulosum
C. lanceolata
G. biloba
T. mairei
C. deodara
W. mirabilis
W. nobilis
G. gnemon
R. piresii
E. equisetina
trnS-UGA
trnY-GUA
trnS-GCU
trnH-GUG
trnO-UUG
trnT-GGU
trnP-UGG
trnL-UAG
trnG-GCC
trnM-CAU
trnD-GUC
trnR-CCG
trnA-UGC
trnC-GCA
trnA-UGC
trnS-CGA
trnR-UCU
trnT-GGU
trnT-UGU
trnO-UUG
trnK-UUU
trnE-UUC
trnI-CAU
trnE-UUC
trnN-GUU
trnN-GUU
trnK-CUU
trnN-GUU
trnT-UGU
trnY-AUA
trnV-UAC
trnI-AAU
trnE-UUC
trnL-CAA
trnL-UAG
trnD-GUC
trnW-CCA
trnM-CAU
trnP-UGG
trnP-GGG
trnR-CCG
trnW-CCA
trnR-CCG
trnR-ACG
trnH-GUG
trnS-GCU
trnS-GGA
trnY-GUA
trnL-UAA
trnI-CAU
trnI-CAU
trnG-GCC
trnT-GGU
trnS-CGA
trnG-UCC
trnI-CAU
trnL-CAA
trnV-GAC
trnI-UAU
trnT-GGU
trnE-UUC
trnK-UUU
trnC-GCA
trnO-UUG
trnH-GUG
trnR-UCU
trnF-GAA
trnA-UGC
trnV-UAC
trnT-UGU
trnM-CAU
represents the presence of one copy of tRNA genes
represents the presence of two copies of tRNA genes
represents the absence of tRNA genes
